# Supplementary material for: NanoCaller for accurate detection of SNPs and indels in difficult-to-map regions from long-read sequencing by haplotype-aware deep neural networks
Source: Genome Biol. 2021 Sep 6;22:261. doi: 10.1186/s13059-021-02472-2 (PMC8419925; doi:10.1186/s13059-021-02472-2)
Supplement: Supplementary file 1 — Additional file 1. : Fig S1-S7 and Tables S1-S26 for various detailed performance evaluation on older Pacbio and Nanopore (basecalled by Guppy 2.3) on GIAB benchmark variants v3.3.2, the definitions of difficult-to-map regions, evaluation with different quality score thresholds, runtime comparisons, discussion of generation of heterozygous sites, long-read statistics, and commands to reproduce performance. [file 13059_2021_2472_MOESM1_ESM.docx]

Additional file 1 of

NanoCaller for accurate detection of SNPs and indels in difficult-to-map regions from long-read sequencing by haplotype-aware deep neural networks

Mian Umair Ahsan ^1,#^, Qian Liu^1,#^, Li Fang, Kai Wang^1,2*^

^1^ Raymond G. Perelman Center for Cellular and Molecular Therapeutics, Children’s Hospital of Philadelphia, Philadelphia, PA 19104, USA

^2^ Department of Pathology and Laboratory Medicine, Perelman School of Medicine, University of Pennsylvania, Philadelphia, PA 19104, USA

^#^ These authors contributed equally to this work.

^*^ To whom correspondence should be addressed. Email: [wangk@email.chop.edu](mailto:wangk@email.chop.edu)

# Statistics of long-reads datasets

Table S1. Whole genome statistics of datasets on five human genomes by Nanopore and PacBio sequencing. Each genome is aligned to the GRCh38 reference genome, and only the mapped reads are used to calculate the statistics. Total number of bases is calculated as the sum of the length of all mapped reads, and the coverage is defined as the number of mapped bases divided by the reference genome length.’Mean’ and ‘Median’ represent the mean and median of read length in long reads for a genome. ONT HG001 reads were basecalled by Guppy 2.3.8, ONT HG002-4 reads were basecalled by Guppy 3.6, and HX1 reads were basecalled with Albacore. HG001 CCS (11kb library size) reads were obtained GIAB’s database, whereas HG002-4 CCS (15kb library size) reads were obtained from precisionFDA truth challenge V2.

| Platform | Genome | # reads | # bases | N50 | Mean | Median | Coverage |
| --- | --- | --- | --- | --- | --- | --- | --- |
| ONT | HG001 | 15,666,888 | 132.9 Gb | 13,630 | 8,485 | 5,387 | 43X |
| ONT | HG002 | 7,788,089 | 144.2 Gb | 52,549 | 18,514 | 6,672 | 49X |
| ONT | HG003 | 13,601,716 | 249.4 Gb | 46,163 | 18,335 | 8,168 | 85X |
| ONT | HG004 | 12,530,839 | 251.3 Gb | 49,563 | 20,051 | 8,371 | 88X |
| ONT | HX1 | 20,497,769 | 271.8 Gb | 22,273 | 13,261 | 10,123 | 88X |
| PacBio (CLR) | HG001 | 23,808,790 | 106.3 Gb | 6,309 | 4,466 | 3,180 | 37X |
| PacBio (CLR) | HG002 | 21,511,145 | 179.0 Gb | 11,264 | 8,323 | 7,500 | 58X |
| PacBio (CLR) | HG003 | 10,564,465 | 85.3 Gb | 10,943 | 8,078 | 7,251 | 28X |
| PacBio (CLR) | HG004 | 10,369,228 | 83.4 Gb | 10,869 | 8,040 | 7,545 | 27X |
| PacBio (CCS) | HG001 | 8,487,716 | 84.6 Gb | 10,004 | 9,963 | 9,973 | 29X |
| PacBio (CCS) | HG002 | 7,979,372 | 102.6 Gb | 12,883 | 12,855 | 12,781 | 36X |
| PacBio (CCS) | HG003 | 6,872,414 | 100.5 Gb | 14,761 | 14,627 | 14,627 | 35X |
| PacBio (CCS) | HG004 | 6,578,333 | 98.9 Gb | 15,100 | 15,031 | 14,949 | 34X |

# Performance of NanoCaller and other variant callers on old Nanopore datasets of the Ashkenazim trio

From Table S2-S5, three NanoCaller models are evaluated: NanoCaller SNP models NanoCaller1 (trained on ONT HG001 Guppy 2.3.8) and NanoCaller2 (trained on ONT HG002 Guppy 2.3.4), and NanoCaller1 indel model trained on ONT HG001 Guppy 2.3.8 reads. All models are trained using GIAB v3.3.2 benchmark variants. From Table S2-S5, the testing is done on HG002 ONT reads basecalled with Guppy 2.3.4, and HG003-4 ONT reads basecalled with Guppy 3.2.

Table S2. Performances of SNP predictions of NanoCaller1 and NanoCaller2 SNP models, along with existing variant callers, on ONT data of the Ashkenazim trio, evaluated against v3.3.2 of GIAB benchmark variants.

|  | HG002 | | | HG003 | | | HG004 | | |
| --- | --- | --- | --- | --- | --- | --- | --- | --- | --- |
|  | Precision | Recall | F1 | Precision | Recall | F1 | Precision | Recall | F1 |
| NanoCaller1 | 97.99 | 96.55 | 97.27 | 97.76 | 96.51 | 97.14 | 97.61 | 96.33 | 96.97 |
| NanoCaller2 | 97.84 | 96.40 | 97.12 | 97.77 | 96.27 | 97.02 | 97.60 | 96.12 | 96.86 |
| Medaka | 97.75 | 96.92 | 97.34 | 97.86 | 96.89 | 97.38 | 97.73 | 96.67 | 97.20 |
| Clair | 98.85 | 96.67 | 97.75 | 99.02 | 97.30 | 98.16 | 98.97 | 97.04 | 98.00 |
| Longshot | 99.05 | 96.53 | 97.78 | 99.13 | 95.16 | 97.11 | 99.05 | 94.98 | 96.98 |

Table S3. Performances of SNP predictions of NanoCaller1 and NanoCaller2 SNP models, along with existing variant callers, on ONT data of the Ashkenazim trio, evaluated against v4.2 of GIAB benchmark variants.

|  | HG002 | | | HG003 | | | HG004 | | |
| --- | --- | --- | --- | --- | --- | --- | --- | --- | --- |
|  | Precision | Recall | F1 | Precision | Recall | F1 | Precision | Recall | F1 |
| NanoCaller1 | 98.90 | 96.77 | 97.83 | 99.03 | 96.77 | 97.89 | 98.96 | 96.50 | 97.72 |
| NanoCaller2 | 98.79 | 96.63 | 97.70 | 98.87 | 96.75 | 97.80 | 98.85 | 96.47 | 97.65 |
| Medaka | 98.81 | 96.98 | 97.89 | 98.95 | 97.04 | 97.99 | 99.02 | 96.84 | 97.92 |
| Clair | 99.01 | 96.61 | 97.80 | 99.07 | 97.51 | 98.29 | 99.09 | 97.24 | 98.16 |
| Longshot | 98.97 | 95.89 | 97.41 | 99.11 | 94.60 | 96.81 | 99.13 | 94.32 | 96.67 |

Table S4. Performances of indel predictions in non-homopolymer regions of NanoCaller, along with existing variant callers, on ONT data of the Ashkenazim trio, evaluated against v3.3.2 of GIAB benchmark variants.

|  | HG002 | | | HG003 | | | HG004 | | |
| --- | --- | --- | --- | --- | --- | --- | --- | --- | --- |
|  | Precision | Recall | F1 | Precision | Recall | F1 | Precision | Recall | F1 |
| NanoCaller1 | 77.37 | 59.65 | 67.37 | 75.90 | 60.09 | 67.08 | 73.56 | 58.30 | 65.05 |
| Medaka | 76.56 | 63.64 | 69.51 | 78.27 | 65.98 | 71.61 | 77.13 | 63.90 | 69.90 |
| Clair | 84.92 | 55.26 | 66.96 | 85.39 | 58.53 | 69.46 | 85.76 | 56.48 | 68.11 |

Table S5. Performances of indel predictions in non-homopolymer regions of NanoCaller, along with existing variant callers, on ONT data of the Ashkenazim trio, evaluated against v4.2 of GIAB benchmark variants.

|  | HG002 | | | HG003 | | | HG004 | | |
| --- | --- | --- | --- | --- | --- | --- | --- | --- | --- |
|  | Precision | Recall | F1 | Precision | Recall | F1 | Precision | Recall | F1 |
| NanoCaller1 | 76.87 | 59.10 | 66.83 | 73.26 | 59.18 | 65.47 | 70.60 | 57.30 | 63.26 |
| Medaka | 76.34 | 62.62 | 68.81 | 77.78 | 65.67 | 71.22 | 76.74 | 63.77 | 69.66 |
| Clair | 84.40 | 54.59 | 66.30 | 85.57 | 58.28 | 69.34 | 85.74 | 56.53 | 68.14 |

# SNP performance of NanoCaller and other variant callers in difficult-to-map regions on Nanopore reads of the Ashkenazim trio

BED file sources of different difficult-to-map regions with respect to GRCh38 reference genome:

1. All difficult regions.
   Source: <ftp://ftp-trace.ncbi.nlm.nih.gov/ReferenceSamples/giab/release/genome-stratifications/v2.0/GRCh38/union/GRCh38_alldifficultregions.bed.gz>
2. Low mappability regions in Table S8.
   Source: <ftp://ftp-trace.ncbi.nlm.nih.gov/ReferenceSamples/giab/release/genome-stratifications/v2.0/GRCh38/mappability/GRCh38_lowmappabilityall.bed.gz>
3. Segmental duplications in Table S9.
   Source: <ftp://ftp-trace.ncbi.nlm.nih.gov/ReferenceSamples/giab/release/genome-stratifications/v2.0/GRCh38/SegmentalDuplications/GRCh38_segdups.bed.gz>
4. Tandem and homopolymer repeats (perfect homopolymers longer than 6bp and imperfect homopolymers longer than 10bp) in Table S10.
   Source: <ftp://ftp-trace.ncbi.nlm.nih.gov/ReferenceSamples/giab/release/genome-stratifications/v2.0/GRCh38/LowComplexity/GRCh38_AllTandemRepeatsandHomopolymers_slop5.bed.gz>
5. Perfect homopolymers longer than 6bp and imperfect homopolymers longer than 10bp.
   Source: <https://ftp-trace.ncbi.nlm.nih.gov/ReferenceSamples/giab/release/genome-stratifications/v2.0/GRCh38/LowComplexity/GRCh38_AllHomopolymers_gt6bp_imperfectgt10bp_slop5.bed.gz>
6. Perfect homopolymers of lengths 4-6bp.
   Source: <https://ftp-trace.ncbi.nlm.nih.gov/ReferenceSamples/giab/release/genome-stratifications/v2.0/GRCh38/LowComplexity/GRCh38_SimpleRepeat_homopolymer_4to6_slop5.bed.gz>
7. Table S11.
   Source: <ftp://ftp-trace.ncbi.nlm.nih.gov/ReferenceSamples/giab/release/genome-stratifications/v2.0/GRCh38/OtherDifficult/GRCh38_MHC.bed.gz>

The details of these files are provided here: <https://ftp-trace.ncbi.nlm.nih.gov/ReferenceSamples/giab/release/genome-stratifications/v2.0/GRCh38/LowComplexity/v2.0-GRCh38-LowComplexity-README.txt>

**Various difficult-to-map regions**

The following difficult-to-map regions are used for performance evaluation and comparison: 1) “all difficult-to-map” regions, 2) low mappability regions, 3) segmental duplications, 4) tandem and homopolymer repeats, and 5) MHC. For each of these categories, we get the evaluation regions by intersecting the corresponding BED file with high-confidence intervals for each genome using BEDtools.

**Easy genomic regions**

Easy genomic regions are also used during performance comparison and are defined as the complement of “all difficult-to-map” regions (GRCh38_alldifficultregions.bed). Easy genomic regions are obtained by removing “all difficult-to-map” regions from each genome’s high-confidence intervals.

**Definition of homopolymer and non-homopolymer regions for indel performance evaluation**

GIAB genome stratification BED is used to obtain homopolymer regions and non-homopolymer regions for evaluating indel performance. Homopolymer regions are obtained by combining GRCh38_AllHomopolymers_gt6bp_imperfectgt10bp_slop5.bed and GRCh38_SimpleRepeat_homopolymer_4to6_slop5.bed BED files. These first BED file contains intervals with perfect homopolymer regions longer than 6bp and imperfect homopolymers longer than 10bp (defined by GIAB as a ‘single base was repeated >10bp except for a 1bp interruption by a different base’, e.g. AAAATAAAAAA). The second BED file contains all intervals of homopolymer regions of lengths between 4 to 6bp. *It is important to note that GRCh38_AllTandemRepeatsandHomopolymers_slop5.bed contains homopolymer regions from the first BED file only, and does not contain homopolymers of lengths 4-6bp from the second BED file*.

Meanwhile, non-homopolymer regions are obtained by subtracting the homopolymer regions from GIAB high-confidence intervals for each genome.

**Table S6. Statistics of number ground truth variants in GIAB v4.2 benchmark of the Ashkenazim trio within various difficult genomic regions identified in GIAB genome stratification v2.0. Numbers shown below are for SNPs, with the exception of MHC region for which both SNP and indel counts are shown.**

| Region | HG002 | HG003 | HG004 |
| --- | --- | --- | --- |
| All Difficult Regions | 628,157 | 611,270 | 619,719 |
| Tandem and Homopolymer Repeats | 182,901 | 166,398 | 166,807 |
| Segmental Duplications | 121,179 | 122,393 | 125,711 |
| Low Mappability regions | 192,749 | 193,153 | 195,332 |
| MHC (SNPs) | 20,139 | 19,730 | 20,576 |
| MHC (indels) | 1667 | 1655 | 1738 |

Please note that in tables S7-S11, the performance of NanoCaller1 model is tested on precisionFDA challenge HG002-4 ONT reads basecalled with Guppy3.6.

Table S7. Performances of SNP predictions in all difficult genomic regions by NanoCaller1 SNP models, along with existing variant callers on ONT data, using 4.2 benchmark variants.

|  |  | **NanoCaller1** | **Medaka** | **Clair** | **Longshot** |
| --- | --- | --- | --- | --- | --- |
|  | Threshold | 144 | 3 | 370 | 118 |
| Precision | HG002 | 96.16 | 94.74 | 94.68 | 97.48 |
|  | HG003 | 97.30 | 96.14 | 96.47 | 96.84 |
|  | HG004 | 97.23 | 96.41 | 96.23 | 96.87 |
| Recall | HG002 | 95.04 | 95.48 | 95.33 | 91.12 |
|  | HG003 | 96.27 | 96.54 | 96.11 | 92.34 |
|  | HG004 | 96.14 | 96.12 | 96.22 | 92.06 |
| F1 | HG002 | **95.60** | 95.11 | 95.01 | 94.19 |
|  | HG003 | **96.78** | 96.34 | 96.29 | 94.53 |
|  | HG004 | **96.68** | 96.27 | 96.22 | 94.40 |

|  |  | **NanoCaller1** | **Medaka** | **Clair** | **Longshot** |
| --- | --- | --- | --- | --- | --- |
|  | Threshold | 200 | 8 | 713 | 172 |
| Precision | HG002 | 96.73 | 95.33 | 95.9 | 98.70 |
|  | HG003 | 96.84 | 95.27 | 96.69 | 97.42 |
|  | HG004 | 96.86 | 96.03 | 96.52 | 97.66 |
| Recall | HG002 | 97.31 | 97.59 | 97.43 | 88.13 |
|  | HG003 | 98.32 | 98.46 | 98.09 | 90.42 |
|  | HG004 | 98.28 | 98.38 | 98.26 | 90.78 |
| F1 | HG002 | **97.02** | 96.45 | 96.66 | 93.12 |
|  | HG003 | **97.57** | 96.84 | 97.39 | 93.79 |
|  | HG004 | **97.57** | 97.19 | 97.38 | 94.09 |

Table S8. Performances of SNP predictions in low mappability regions by NanoCaller1 SNP models, along with existing variant callers on ONT data, using 4.2 benchmark variants.

|  |  | **NanoCaller1** | **Medaka** | **Clair** | **Longshot** |
| --- | --- | --- | --- | --- | --- |
|  | Threshold | 228 | 11 | 768 | 186 |
| Precision | HG002 | 94.19 | 90.79 | 93.07 | 97.99 |
|  | HG003 | 94.43 | 92.2 | 94.55 | 95.86 |
|  | HG004 | 94.58 | 93.64 | 94.3 | 96.30 |
| Recall | HG002 | 95.64 | 96.54 | 96.23 | 84.99 |
|  | HG003 | 97.19 | 97.46 | 97.16 | 88.9 |
|  | HG004 | 97.17 | 97.31 | 97.27 | 89.27 |
| F1 | HG002 | **94.91** | 93.58 | 94.62 | 91.03 |
|  | HG003 | 95.79 | 94.75 | **95.84** | 92.25 |
|  | HG004 | **95.86** | 95.44 | 95.76 | 92.66 |

Table S9. Performances of SNP predictions in segmental duplication regions by NanoCaller1 SNP models, along with existing variant callers on ONT data, using 4.2 benchmark variants.

Table S10. Performances of SNP predictions in tandem and homopolymer repeat regions by NanoCaller1 SNP models, along with existing variant callers on ONT data, using 4.2 benchmark variants.

|  |  | **NanoCaller1** | **Medaka** | **Clair** | **Longshot** |
| --- | --- | --- | --- | --- | --- |
|  | Threshold | 93 | 2 | 300 | 115 |
| Precision | HG002 | 93.20 | 90.09 | 90.48 | 93.96 |
|  | HG003 | 95.48 | 93.49 | 93.38 | 93.23 |
|  | HG004 | 95.20 | 92.63 | 92.85 | 92.70 |
| Recall | HG002 | 88.53 | 88.30 | 89.05 | 89.67 |
|  | HG003 | 90.30 | 90.14 | 90.29 | 92.31 |
|  | HG004 | 89.78 | 89.57 | 90.01 | 91.81 |
| F1 | HG002 | 90.81 | 89.19 | 89.76 | **91.76** |
|  | HG003 | **92.82** | 91.78 | 91.81 | 92.76 |
|  | HG004 | **92.41** | 91.07 | 91.41 | 92.25 |

Table S11. Performances of SNPs, indels and overall variants predictions in MHC by NanoCaller1 SNP models, along with existing variant callers on ONT data, using 4.2 benchmark variants. Overall variant performance is calculated by combining the SNPs and indels performances. The ensemble variant calls are created by combining NanoCaller1, Medaka and Clair variant calls and was submitted to precisionFDA Truth Challenge V2.

| **SNPs Performance** | | | | | | |
| --- | --- | --- | --- | --- | --- | --- |
|  |  | Ensemble | NanoCaller1 | Medaka | Clair | Longshot |
|  | Threshold | -- | 145 | 0 | 272 | 15 |
| Precision | HG002 | 99.44 | 98.88 | 99.02 | 97.88 | 98.77 |
|  | HG003 | 99.58 | 99.37 | 99.61 | 98.76 | 99.10 |
|  | HG004 | 99.51 | 99.18 | 97.92 | 98.77 | 99.00 |
| Recall | HG002 | 98.50 | 98.18 | 95.34 | 97.23 | 52.09 |
|  | HG003 | 99.11 | 99.09 | 99.23 | 98.60 | 57.24 |
|  | HG004 | 98.84 | 98.97 | 90.93 | 98.40 | 51.05 |
| F1 | HG002 | 98.97 | **98.53** | 97.15 | 97.55 | 68.21 |
|  | HG003 | 99.34 | 99.23 | **99.42** | 98.68 | 72.57 |
|  | HG004 | 99.17 | **99.07** | 94.29 | 98.59 | 67.37 |
| **Indels Performance** | | | | | | |
|  |  | Ensemble | NanoCaller1 | Medaka | Clair | Longshot |
|  | Threshold | -- | 205 | 19 | 509 | -- |
| Precision | HG002 | 79.40 | 72.77 | 74.02 | 81.00 | -- |
|  | HG003 | 80.23 | 75.02 | 80.20 | 78.99 | -- |
|  | HG004 | 76.39 | 74.59 | 75.80 | 81.32 | -- |
| Recall | HG002 | 57.21 | 59.47 | 64.78 | 57.31 | -- |
|  | HG003 | 66.18 | 61.89 | 78.47 | 61.59 | -- |
|  | HG004 | 64.00 | 64.55 | 68.84 | 60.86 | -- |
| F1 | HG002 | 66.51 | 65.45 | **69.09** | 67.13 | -- |
|  | HG003 | 72.53 | 67.82 | **79.33** | 69.21 | -- |
|  | HG004 | 69.64 | 69.21 | **72.15** | 69.62 | -- |
| **Overall Variants Performance** | | | | | | |
|  |  | Ensemble | NanoCaller1 | Medaka | Clair | Longshot |
| Precision | HG002 | 98.30 | 97.20 | 97.19 | 96.92 | -- |
|  | HG003 | 98.30 | 97.78 | 98.06 | 97.51 | -- |
|  | HG004 | 97.94 | 97.48 | 96.16 | 97.70 | -- |
| Recall | HG002 | 95.34 | 95.22 | 92.99 | 94.18 | -- |
|  | HG003 | 96.56 | 96.21 | 97.62 | 95.73 | -- |
|  | HG004 | 96.12 | 96.28 | 89.20 | 95.46 | -- |
| F1 | HG002 | 96.79 | **96.20** | 95.05 | 95.53 | -- |
|  | HG003 | 97.42 | 96.99 | **97.84** | 96.61 | -- |
|  | HG004 | 97.02 | **96.87** | 92.55 | 96.57 | -- |

# Novel variants validated by Sanger sequencing

Table S12. Sanger validated variants, and performance of predictions in HG002 genome by various variant callers using HG002 ONT reads basecalled with Guppy 3.6. These variants are missing in GIAB v3.3.2 of HG002.

| Chrom | Position | REF | ALT | Genotype | Nanocaller | Medaka | Clair | Longshot |
| --- | --- | --- | --- | --- | --- | --- | --- | --- |
| chr1 | 78883942 | C | T | 1\|0 | Correct | Incorrect variant type | Correct | -- |
| chr1 | 78883952 | ATATATATTTATCCTTTATATATATATTCTT | A | 1\|0 | Incorrect allele | Incorrect allele | -- | -- |
| chr2 | 227913871 | A | ATATCTATCTATC | 1\|0 | Correct | Correct | Correct | -- |
| chr2 | 227913885 | G | A | 1\|0 | Correct | Correct | Correct | Correct |
| chr2 | 227913889 | G | A | 1\|0 | Correct | Correct | Correct | Correct |
| chr2 | 227913928 | T | TA | 1\|0 | Correct | Correct | Correct | -- |
| chr2 | 227913931 | A | T | 1\|0 | Correct | Correct | Correct | Correct |
| chr3 | 5336450 | A | T,C | 1\|2 | Incorrect allele and zygosity | Incorrect allele and zygosity | Incorrect allele and zygosity | Incorrect allele and zygosity |
| chr3 | 5336452 | C | CGCGT | 0\|1 | -- | -- | -- | -- |
| chr3 | 5336465 | ACACACACACACG | A | 0\|1 | -- | -- | -- | -- |
| chr3 | 5336477 | GCA | G | 1\|0 | Correct | Incorrect allele | Incorrect allele | -- |
| chr3 | 5336487 | A | G | 0\|1 | Incorrect variant  type | -- | Correct | Correct |
| chr6 | 160009985 | C | CTTAA | 0\|1 | Incorrect allele | Incorrect allele | Correct | -- |
| chr6 | 160009986 | C | A | 0\|1 | Incorrect zygosity | Incorrect allele | Incorrect variant type | Incorrect zygosity |
| chr6 | 167130970 | G | GGGCCCCCCTCCCTCCGGGACTCCTCCCTCT | 0\|1 | -- | -- | -- | -- |
| chr6 | 167130972 | GA | G | 1\|0 | Correct | Correct | Correct | -- |
| chr6 | 167130973 | A | G | 0\|1 | Incorrect allele and zygosity | Incorrect allele | Correct | Incorrect allele and zygosity |
| chr6 | 167130976 | A | C | 1\|1 | Correct | Correct | Correct | Correct |
| chr6 | 167130986 | T | C | 1\|0 | Correct | Correct | Incorrect variant type | Correct |
| chr6 | 167130989 | A | G | 1\|1 | Correct | Correct | Correct | Correct |
| chr6 | 167130990 | C | A | 1\|0 | Correct | Correct | -- | Correct |
| chr6 | 167130992 | C | T | 1\|0 | Correct | -- | -- | Correct |
| chr9 | 134784949 | C | T | 0\|1 | Correct | Incorrect variant type | -- | Correct |
| chr9 | 134784951 | G | T | 0\|1 | -- | -- | -- | -- |
| chr9 | 134784955 | G | GGGGGGCA | 0\|1 | Incorrect allele | -- | Incorrect allele | -- |
| chr9 | 134784956 | T | G | 0\|1 | Correct | Incorrect variant type | Incorrect variant type | Correct |
| chr9 | 135663795 | ACAGAGGGGGACCTGGAGGGGCAGAGGAGAGACCTGTGGGG | A | 0\|1 | -- | Correct | -- | -- |
| chr9 | 135663892 | A | G | 1\|1 | Correct | Correct | Correct | Correct |
| chr9 | 135663893 | T | A,G | 1\|2 | Correct | Correct | Correct | Incorrect allele and zygosity |
| chr11 | 113466435 | G | GC | 1\|1 | Correct | Correct | Correct | -- |
| chr11 | 113466437 | A | T | 1\|1 | Correct | Correct | Incorrect variant type | Correct |
| chr12 | 100940063 | A | AT | 0\|1 | Correct | Correct | Correct | -- |
| chr12 | 100940065 | G | C | 0\|1 | Correct | Correct | Correct | Correct |
| chr14 | 75318035 | C | T | 1\|0 | Correct | Correct | Correct | Correct |
| chr14 | 75318038 | AT | A | 1\|0 | Incorrect allele | Incorrect allele | Correct | -- |
| chr14 | 75318052 | T | C | 1\|0 | Correct | -- | Correct | Correct |
| chr14 | 75318054 | T | G | 1\|0 | Correct | Correct | Correct | Correct |
| chr20 | 11064571 | T | TGA | 0\|1 | Correct | Correct | -- | -- |
| chr20 | 11064574 | A | ATTTTCAAGACTATTGTGACTATGAC | 0\|1 | Correct | Correct | Correct | -- |
| chr20 | 11064578 | A | T | 0\|1 | Correct | Correct | -- | -- |
| chr20 | 11064579 | C | T | 0\|1 | Correct | Correct | Incorrect variant type | Correct |

# Quality score thresholds for filtering variant calls

Precision/recall/F1 statistics for each variant caller are investigated with respect to the recommended quality score, or by taking average over quality scores giving highest F1-score when evaluated by RTG’s *vcfeval*. Here the quality thresholds for different Nanopore models on various datasets can show how the quality of reads, depth and quality of ground truth variants affect the optimal performance evaluation. In summary, the range of SNP quality scores is 30-999, but Table S13 shows that SNP F1-scores of both NanoCaller1 and NanoCaller2 SNP models are very resilient to small changes on quality score thresholds. For NanoCaller1 and NanoCaller3 SNP models, the best performance on PacBio CCS reads can be obtained without any specialized threshold (which corresponds to a quality score threshold of 30). For PacBio CLR reads, genome coverage significantly effects SNP calling performance, thus we choose different thresholds for HG001/HG002 and HG003/HG004, as shown in Table S14.

Please note in Tables S13-S16, NanoCaller models are tested on precisionFDA challenge HG002-4 ONT reads basecalled with Guppy3.6, HG001 ONT reads basecalled with Guppy 2.3.8, HX1 ONT reads basecalled with Albacore, HG001 CCS (11kb library size) reads obtained GIAB’s database, and HG002-4 CCS (15kb library size) reads downloaded from precisionFDA truth challenge V2.

Table S13. F1 scores of NanoCaller1 and NanoCaller2 SNP models on ONT datasets. 162 and 78 are recommended as quality thresholds for NanoCaller1 and NanoCaller2 models, respectively. The column ‘Quality Score Range’ shows the range of quality scores that give the optimal SNP F1-score shown in the ‘Best F1’ column. Third colum under each model shows the F1-score with the recommended threshold of quality scores.

|  | NanoCaller1 | | | NanoCaller2 | | |
| --- | --- | --- | --- | --- | --- | --- |
|  | Quality Score Range | Best F1 | F1 with Quality Score=162 | Quality Score Range | Best F1 | F1 with Quality Score=78 |
| HG001 | 120-120 | 95.54 | 95.40 | 65-70 | 94.93 | 94.89 |
| HG002 | 179-201 | 98.03 | 97.99 | 99-117 | 98.03 | 97.97 |
| HG003 | 157-185 | 98.88 | 98.88 | 62-85 | 98.88 | 98.88 |
| HG004 | 166-191 | 98.78 | 98.77 | 71-87 | 98.82 | 98.82 |
| HX1 | 191-194 | 90.52 | 90.28 | 93-97 | 91.07 | 90.94 |

Table S14. F1 scores of NanoCaller1 and NanoCaller3 SNP models on CLR datasets. The thresholds of quality scores are recommended for higher coverage genomes HG001/HG002 and lower coverage genomes HG003/HG004. The column ‘Quality Score Range’ shows the range of quality scores that give the optimal SNP F1-score shown in the ‘Best F1’ column. Third and fourth columns under each model shows the F1-score with the recommended thresholds of quality scores.

|  | NanoCaller1 | | |  | NanoCaller3 | | |  |
| --- | --- | --- | --- | --- | --- | --- | --- | --- |
|  | Quality Score Range | Best F1 | Reported Quality Cut-off | F1 score using reported Cut-off | Quality Score Range | Best F1 | Reported Quality Cut-off | F1 score using reported Cut-off |
| HG001 | 30 | 93.97 | None | 93.97 | 30-37 | 94.95 | 55 | 94.90 |
| HG002 | 32-34 | 98.26 | None | 98.26 | 79-111 | 98.29 | 55 | 98.24 |
| HG003 | 108-114 | 94.33 | 110 | 94.33 | 202-210 | 93.53 | 210 | 93.53 |
| HG004 | 118-132 | 93.49 | 110 | 93.47 | 209-218 | 92.61 | 210 | 92.59 |

Table S15. F1 scores of NanoCaller ONT and PacBio indel models. 44 and 25 are recommended as quality thresholds for ONT and PacBio models, respectively. The column ‘Quality Score Range’ shows the range of quality scores that give the optimal indel F1-score shown in the ‘Best F1’ column. Third colum under each model shows the F1-score with the recommended thresholds of quality scores.

|  | ONT model | | | PacBio model | | |
| --- | --- | --- | --- | --- | --- | --- |
|  | Quality Score Range | Best F1 | F1 with Quality Score=144 | Quality Score Range | Best F1 | F1 with Quality Score=25 |
| HG001 | 243-247 | 56.79 | 53.30 | 2-8 | 94.16 | 94.09 |
| HG002 | 145-152 | 79.75 | 79.74 | 2-5 | 94.52 | 94.40 |
| HG003 | 101-101 | 81.31 | 81.23 | 52-55 | 93.68 | 93.53 |
| HG004 | 101-104 | 79.76 | 79.71 | 56-64 | 93.61 | 93.37 |

Table S16. Quality thresholds used for different variant callers for different sequencing technologies. For Clair and PacBio, the developer recommended thresholds are used, whereas DeepVariant developers do not recommend using any threshold. For Medaka, the average of best quality scores for SNPs over five ONT genomes are calculated, and used as final SNP quality cut-off (the same procedure for the quality thresholds of indels). For WhatsHap, best results are obtained without any quality cut-off as well.

| Variant Caller | Sequencing Type | Threshold |
| --- | --- | --- |
| Medaka | ONT | 2 (SNPs), 23 (indels) |
| Clair | ONT | 748 |
|  | PacBio | 113 |
| Longshot | ONT | 65 |
|  | PacBio | 50 |
| WhatsHap | ONT, PacBio | None |
| DeepVariant | PacBio | None |

Runtime Comparisons

Table 17. Wall-clock runtimes for various variant callers using Intel Xeon CPU E5-2683 v4 @ 2.10GHz. For variant callers that support parallelization, 16 CPUs are used. The runtime evaluation is tested on 49X HG002 ONT reads basecalled with Guppy 3.6, 35X HG002 PacBio CCS reads (15kb library size) from precisionFDA challenge, and 58X HG002 PacBio CLR reads.

|  | Variant Caller | Time (hours) | Types of variants | Number of CPUs | Parallelization Supported |
| --- | --- | --- | --- | --- | --- |
| ONT 49X | NanoCaller | 18.4 | SNPs and Indels | 16 | Yes |
|  | Medaka | 181.6 | SNPs and Indels | 16 | Yes |
|  | Clair | 5.6 | SNPs and Indels | 16 | Yes |
|  | Longshot | 49.7 | SNPs | 1 | No |
|  | WhatsHap | 84.3 | SNPs | 1 | No |
| CCS 35X | NanoCaller | 11.2 | SNPs and Indels | 16 | Yes |
|  | Clair | 1.8 | SNPs and Indels | 16 | Yes |
|  | Longshot | 14.4 | SNPs | 1 | No |
|  | WhatsHap | 57.3 | SNPs | 1 | No |
|  | DeepVariant | 11.8 | SNPs and Indels | 16 | Yes |
| CLR 58X | NanoCaller | 4.8 | SNPs | 16 | Yes |
|  | Clair | 3.2 | SNPs and Indels | 16 | Yes |
|  | Longshot | 11.1 | SNPs | 1 | No |

Selection of nearby potentially heterozygous sites for SNP calling feature generation

Please note that in Tables S18-S23, NanoCaller models are evaluated on precisionFDA challenge HG002-4 ONT reads basecalled with Guppy3.6, HG002-4 CCS (15kb library size) datasets from precisionFDA truth challenge V2, HG002-4 PacBio CLR reads.

Table S18. Number of potentially heterozygous Sites chosen for each candidate site under two methods. Method 1 is used for SNP calling on ONT reads and selects the given number of sites from each range given. Method 2 is used for SNP calling on PacBio reads. In each range for either method, the specified number of sites closest to the candidate site are selected, because they share more reads with the candidate site. The design of these two methods is motivated by the difference in read length distribution of ONT and PacBio reads, shown in Fig S2. An illustration of the two methods is shown in Fig S3.

| Method 1 (used for SNP calling on ONT reads) | | Method 2 (used for SNP calling on PacBio reads) | |
| --- | --- | --- | --- |
| Range | Number of Potentially Heterozygous Sites Chosen | Range | Number of Potentially Heterozygous Sites Chosen |
| -50kbp to -20kbp | 6 |  |  |
| -20kbp to -10kbp | 5 | -20kbp to 0 | 20 |
| -10kbp to -5kbp | 4 |  |  |
| -5 kbp to -2kbp | 3 |  |  |
| -2kbp to 0 | 2 |  |  |
| SNP Candidate Site |  | SNP Candidate Site |  |
| 0 to +2kbp | 2 | 0 to +20kbp | 20 |
| +2kbp to +5kbp | 3 |  |  |
| +5kbp to +10kbp | 4 |  |  |
| +10kbp to +20kbp | 5 |  |  |
| +20kbp to +50kbp | 6 |  |  |

Table S19. Performance of NanoCaller SNP calling on HG004 ONT, CCS and CLR reads with various thresholds used to define heterozygous SNPs. The best F1-scores with their own thresholds on v4.2 benchmark variants are shown here.

| **Oxford Nanopore Reads** | | | |
| --- | --- | --- | --- |
| Threshold | Precision | Recall | F1 |
| 0.30-0.60 | 0.9846 | 0.9843 | 0.9845 |
| 0.30-0.70 | 0.9840 | 0.9833 | 0.9837 |
| 0.30-0.80 | 0.9814 | 0.9797 | 0.9806 |
| 0.35-0.65 | 0.9878 | 0.9857 | 0.9868 |
| 0.40-0.60 | 0.9888 | 0.9867 | **0.9878** |
| 0.40-0.70 | 0.9882 | 0.9847 | 0.9865 |
| 0.40-0.80 | 0.9845 | 0.9790 | 0.9818 |
| 0.45-0.55 | 0.9880 | 0.9851 | 0.9866 |
| **PacBio CLR Reads** | | | |
| Threshold | Precision | Recall | F1 |
| 0.20-0.70 | 0.9527 | 0.8829 | 0.9165 |
| 0.30-0.60 | 0.9595 | 0.9114 | **0.9349** |
| 0.30-0.70 | 0.9559 | 0.8971 | 0.9256 |
| 0.30-0.80 | 0.9410 | 0.8730 | 0.9058 |
| 0.35-0.65 | 0.9534 | 0.8989 | 0.9254 |
| 0.40-0.60 | 0.9457 | 0.8906 | 0.9174 |
| 0.40-0.70 | 0.9409 | 0.8689 | 0.9035 |
| 0.40-0.80 | 0.9215 | 0.8400 | 0.8789 |
| 0.45-0.55 | 0.9228 | 0.8607 | 0.8907 |
| **PacBio CCS Reads** | | | |
| Threshold | Precision | Recall | F1 |
| 0.30-0.60 | 0.9920 | 0.9944 | 0.9932 |
| 0.30-0.70 | 0.9936 | 0.9963 | **0.9950** |
| 0.30-0.80 | 0.9935 | 0.9967 | 0.9951 |
| 0.35-0.65 | 0.9932 | 0.9950 | 0.9941 |
| 0.40-0.60 | 0.9900 | 0.9899 | 0.9900 |
| 0.40-0.70 | 0.9936 | 0.9950 | 0.9943 |
| 0.40-0.80 | 0.9935 | 0.9957 | 0.9946 |
| 0.45-0.55 | 0.9769 | 0.9702 | 0.9735 |

Table S20. Performance of NanoCaller SNP calling on HG004 ONT and CCS reads with various thresholds for minimum number (first column) of heterozygous SNPs required for a a candidate site. The best F1-scores with their own thresholds on v4.2 benchmark variants are shown here.

| **Oxford Nanopore Reads** | | | |
| --- | --- | --- | --- |
| Minimum Number | Precision | Recall | F1 |
| 1 | 98.88 | 98.67 | 98.78 |
| 2 | 98.84 | 98.69 | 98.77 |
| 3 | 98.84 | 98.65 | 98.75 |
| 5 | 98.90 | 98.35 | 98.63 |
| 7 | 98.94 | 97.66 | 98.30 |
| 9 | 98.96 | 96.43 | 97.68 |
| 13 | 98.93 | 92.21 | 95.46 |
| **PacBio CCS** | | | |
| Minimum Number | Precision | Recall | F1 |
| **1** | **99.37** | **99.63** | **99.50** |
| 3 | 99.41 | 95.54 | 97.44 |
| 5 | 99.44 | 90.53 | 94.77 |
| 7 | 99.44 | 87.04 | 92.83 |
| 9 | 99.44 | 84.79 | 91.53 |

Table S21. Performance of NanoCaller SNP calling, in whole genome and various difficult genomic regions, for HG002, HG003 and HG004 ONT reads using Method1 and Method2 described in Table S18. Shown are the best F1-scores achieved by NanoCaller1 or NanoCaller2 SNP models with evaluation on v4.2 benchmark variants.

| NanoCaller1 | | | | | | | |
| --- | --- | --- | --- | --- | --- | --- | --- |
| Genomic Regions | Genomes | Method1 (for ONT reads) | | | Method2 (for PacBio reads) | | |
|  |  | Precision | Recall | F1 | Precision | Recall | F1 |
| Whole genome | HG002 | 98.23 | 97.82 | 98.03 | 98.19 | 97.44 | 97.82 |
|  | HG003 | 99.00 | 98.76 | 98.88 | 98.98 | 98.51 | 98.75 |
|  | HG004 | 98.88 | 98.67 | 98.78 | 98.80 | 98.45 | 98.63 |
| All difficult regions | HG002 | 96.59 | 94.68 | 95.63 | 96.19 | 94.65 | 95.42 |
|  | HG003 | 97.13 | 96.44 | 96.79 | 97.02 | 96.34 | 96.68 |
|  | HG004 | 97.19 | 96.19 | 96.69 | 97.03 | 96.07 | 96.55 |
| Low Mappability Regions | HG002 | 96.63 | 97.40 | 97.02 | 96.61 | 97.14 | 96.88 |
|  | HG003 | 96.77 | 98.37 | 97.57 | 96.80 | 98.18 | 97.49 |
|  | HG004 | 97.00 | 98.15 | 97.58 | 96.80 | 98.14 | 97.47 |
| Tandem and Homopolymer Repeats | HG002 | 93.87 | 88.04 | 90.87 | 92.98 | 88.27 | 90.57 |
|  | HG003 | 95.32 | 90.47 | 92.84 | 95.04 | 90.33 | 92.63 |
|  | HG004 | 94.99 | 89.99 | 92.43 | 94.83 | 89.66 | 92.18 |
| Segmental Duplications | HG002 | 94.07 | 95.81 | 94.94 | 93.83 | 95.72 | 94.77 |
|  | HG003 | 94.65 | 96.99 | 95.81 | 94.51 | 96.97 | 95.73 |
|  | HG004 | 94.51 | 97.26 | 95.87 | 94.41 | 97.20 | 95.79 |
| MHC | HG002 | 98.89 | 98.18 | 98.54 | 98.83 | 98.12 | 98.48 |
|  | HG003 | 99.36 | 99.11 | 99.24 | 99.39 | 98.88 | 99.14 |
|  | HG004 | 99.21 | 98.96 | 99.09 | 99.21 | 98.82 | 99.02 |
| **NanoCaller2** | | | | | | | |
| Genomic Regions | Genomes | Method1 (for ONT reads) | | | Method2 (for PacBio reads) | | |
|  |  | Precision | Recall | F1 | Precision | Recall | F1 |
| Whole genome | HG002 | 98.26 | 97.79 | 98.03 | 98.09 | 97.37 | 97.73 |
|  | HG003 | 99.03 | 98.72 | 98.88 | 98.95 | 98.40 | 98.68 |
|  | HG004 | 98.97 | 98.66 | 98.82 | 98.90 | 98.29 | 98.60 |
| All difficult regions | HG002 | 96.07 | 94.65 | 95.36 | 95.99 | 94.26 | 95.12 |
|  | HG003 | 96.93 | 96.21 | 96.57 | 96.91 | 95.91 | 96.41 |
|  | HG004 | 96.91 | 96.09 | 96.50 | 96.87 | 95.77 | 96.32 |
| Low Mappability Regions | HG002 | 96.11 | 97.17 | 96.64 | 96.01 | 96.86 | 96.44 |
|  | HG003 | 96.18 | 98.21 | 97.19 | 96.26 | 97.90 | 97.08 |
|  | HG004 | 96.24 | 98.25 | 97.24 | 96.27 | 97.88 | 97.07 |
| Tandem and Hoomopolymer Repeats | HG002 | 93.88 | 87.39 | 90.52 | 93.92 | 86.76 | 90.20 |
|  | HG003 | 95.56 | 89.51 | 92.44 | 95.35 | 89.25 | 92.20 |
|  | HG004 | 95.25 | 89.01 | 92.03 | 95.17 | 88.64 | 91.79 |
| Segmental Duplications | HG002 | 92.73 | 95.63 | 94.16 | 92.24 | 95.65 | 93.92 |
|  | HG003 | 93.21 | 97.14 | 95.14 | 93.25 | 96.85 | 95.02 |
|  | HG004 | 93.59 | 97.12 | 95.33 | 93.50 | 96.91 | 95.18 |
| MHC | HG002 | 98.90 | 98.25 | 98.58 | 98.88 | 97.95 | 98.42 |
|  | HG003 | 99.43 | 98.98 | 99.21 | 99.47 | 98.69 | 99.08 |
|  | HG004 | 99.43 | 98.94 | 99.19 | 99.38 | 98.83 | 99.11 |

Table S22. Performance of NanoCaller whole genome SNP calling for HG002, HG003 and HG004 PacBio CCS and CLR reads using Method1 and Method2 described in Table S18. Shown are the best F1-score achieved by NanoCaller1 SNP model with evaluation on v4.2 benchmark variants.

| PacBio CCS Reads | | | | | | | |
| --- | --- | --- | --- | --- | --- | --- | --- |
|  | Genomes | Method1 (for ONT reads) | | | Method2 (for PacBio reads) | | |
|  |  | Precision | Recall | F1 | Precision | Recall | F1 |
| Whole genome | HG002 | 99.27 | 99.53 | 99.40 | 99.31 | 99.60 | 99.45 |
|  | HG003 | 99.33 | 99.56 | 99.45 | 99.36 | 99.63 | 99.49 |
|  | HG004 | 99.33 | 99.57 | 99.45 | 99.37 | 99.63 | 99.50 |
| **PacBio CLR Reads** | | | | | | | |
|  | Genomes | Method1 (for ONT reads) | | | Method2 (for PacBio reads) | | |
|  |  | Precision | Recall | F1 | Precision | Recall | F1 |
| Whole genome | HG002 | 98.68 | 97.73 | 98.20 | 98.55 | 97.98 | 98.27 |
|  | HG003 | 96.15 | 92.47 | 94.28 | 96.46 | 92.28 | 94.33 |
|  | HG004 | 95.80 | 91.15 | 93.42 | 95.95 | 91.14 | 93.49 |

Effects of allowing WhatsHap to change genotype on SNP calling performance

Table S23. Performance of SNP calling by NanoCaller with and without the use of ‘distrust genotypes’ for phasing by WhatsHap. All the other results shown elsewhere besides this table are generated without ‘distrust genotype’ option whiich allows WhatsHap to change genotypes. NanoCaller users can enable ‘distrust genotype’ option by setting ‘enable_whatshap’ flag in NanoCaller run. By default this option is turned off in NanoCaller.

| **Oxford Nanopore Reads** | | | | | | | | | | | | |
| --- | --- | --- | --- | --- | --- | --- | --- | --- | --- | --- | --- | --- |
|  | NanoCaller1 | | | | | | NanoCaller2 | | | | | |
|  | Without ‘enable_whatshap' | | | With ‘enable_whatshap’ | | | Without ‘enable_whatshap’ | | | With ‘enable_whatshap’ | | |
|  | Precision | Recall | F1 | Precision | Recall | F1 | Precision | Recall | F1 | Precision | Recall | F1 |
| HG001 | 96.50 | 94.59 | 95.54 | 96.53 | 94.90 | 95.71 | 96.13 | 93.75 | 94.93 | 96.20 | 94.07 | 95.13 |
| HG002 | 98.23 | 97.82 | 98.03 | 98.24 | 97.87 | 98.06 | 98.26 | 97.79 | 98.03 | 98.19 | 97.92 | 98.06 |
| HG003 | 99.00 | 98.76 | 98.88 | 98.93 | 98.80 | 98.87 | 99.03 | 98.72 | 98.88 | 99.05 | 98.68 | 98.87 |
| HG004 | 98.88 | 98.67 | 98.78 | 98.83 | 98.70 | 98.77 | 98.97 | 98.66 | 98.82 | 98.94 | 98.66 | 98.80 |
| HX1 | 92.61 | 88.51 | 90.52 | 92.05 | 88.41 | 90.20 | 93.39 | 88.85 | 91.07 | 92.89 | 88.89 | 90.85 |
| **PacBio CCS Reads** | | | | | | | | | | | | |
|  | NanoCaller1 | | | | | | NanoCaller3 | | | | | |
|  | Without ‘enable_whatshap' | | | With ‘enable_whatshap’ | | | Without ‘enable_whatshap’ | | | With ‘enable_whatshap’ | | |
|  | Precision | Recall | F1 | Precision | Recall | F1 | Precision | Recall | F1 | Precision | Recall | F1 |
| HG001 | 96.97 | 99.37 | 98.15 | 97.44 | 99.72 | 98.57 | 96.69 | 99.36 | 98.01 | 97.28 | 99.81 | 98.53 |
| HG002 | 99.31 | 99.60 | 99.45 | 99.56 | 99.79 | 99.68 | 99.16 | 99.56 | 99.36 | 99.52 | 99.83 | 99.68 |
| HG003 | 99.36 | 99.63 | 99.49 | 99.58 | 99.78 | 99.68 | 99.22 | 99.58 | 99.40 | 99.54 | 99.82 | 99.68 |
| HG004 | 99.37 | 99.63 | 99.50 | 99.58 | 99.77 | 99.67 | 99.23 | 99.59 | 99.41 | 99.53 | 99.81 | 99.67 |
| **PacBio CLR Reads** | | | | | | | | | | | | |
|  | NanoCaller1 | | | | | | NanoCaller3 | | | | | |
|  | Without ‘enable_whatshap' | | | With ‘enable_whatshap’ | | | Without ‘enable_whatshap’ | | | With ‘enable_whatshap’ | | |
|  | Precision | Recall | F1 | Precision | Recall | F1 | Precision | Recall | F1 | Precision | Recall | F1 |
| HG001 | 97.72 | 90.51 | 93.97 | 98.24 | 90.98 | 94.47 | 96.78 | 93.20 | 94.95 | 97.68 | 94.01 | 95.81 |
| HG002 | 98.55 | 97.98 | 98.27 | 98.78 | 98.25 | 98.51 | 98.11 | 98.46 | 98.29 | 98.17 | 99.09 | 98.63 |
| HG003 | 96.46 | 92.28 | 94.33 | 96.56 | 93.42 | 94.97 | 95.58 | 91.56 | 93.53 | 95.93 | 92.52 | 94.20 |
| HG004 | 95.95 | 91.14 | 93.49 | 96.24 | 92.22 | 94.19 | 94.98 | 90.34 | 92.61 | 95.50 | 91.23 | 93.32 |

Figures

Fig S1. Concordance of ground truth variants correctly predicted by various variant callers on Nanopore reads of the Ashkenazim trio basecalled with Guppy 3.6. Venn diagrams show the overlap of v4.2 ground truth variant calls predicted correctly by NanoCaller, Medaka and Clair. a) SNPs, b) indels. All variants are inside high-confidence regions.


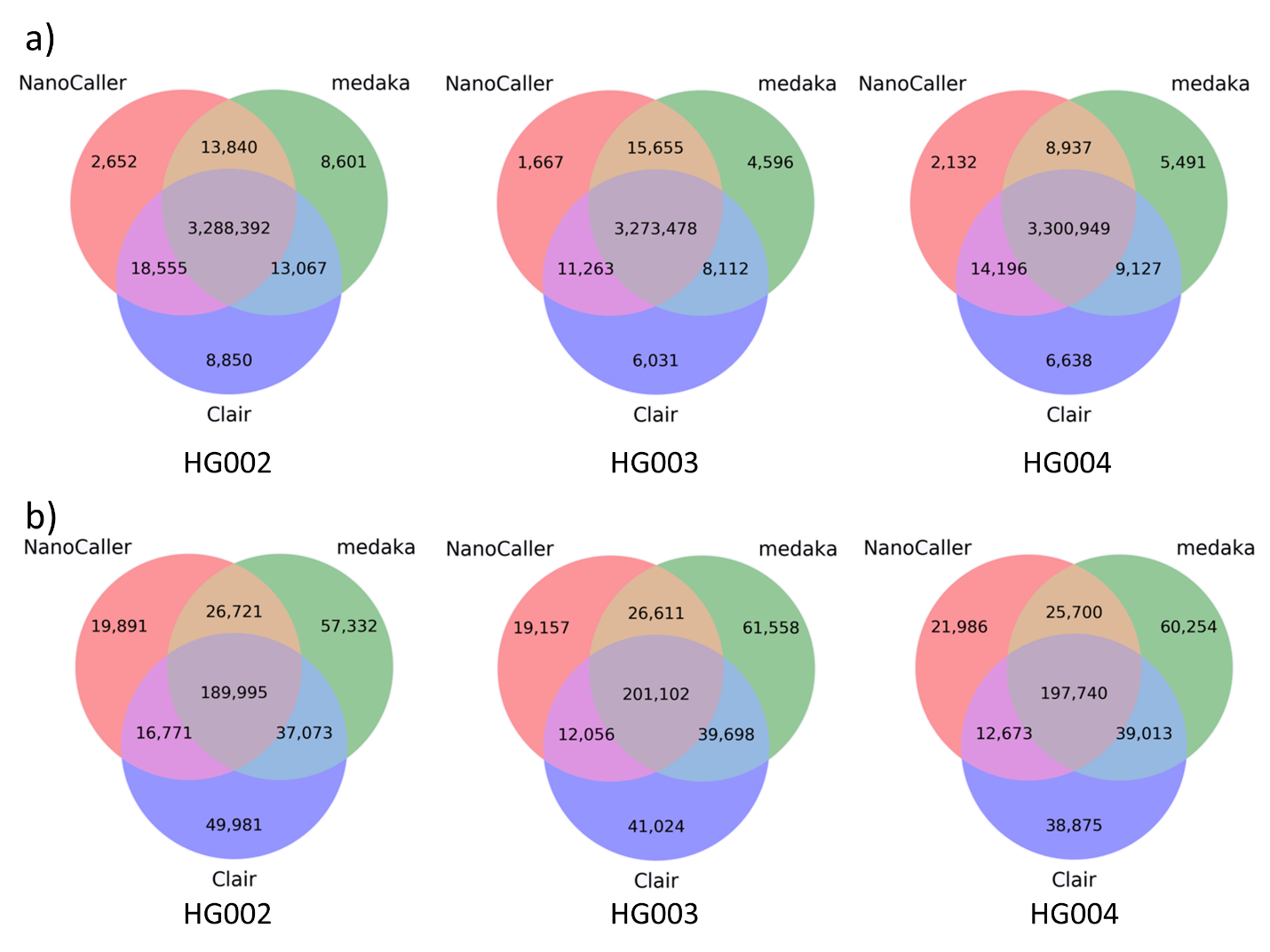


Fig S2. Read length distributions of the following HG004 datasets: 88X ONT reads basecalled by Guppy 3.6, 35X PacBio CCS reads (library size 15kb) and 27X PacBio CLR reads.


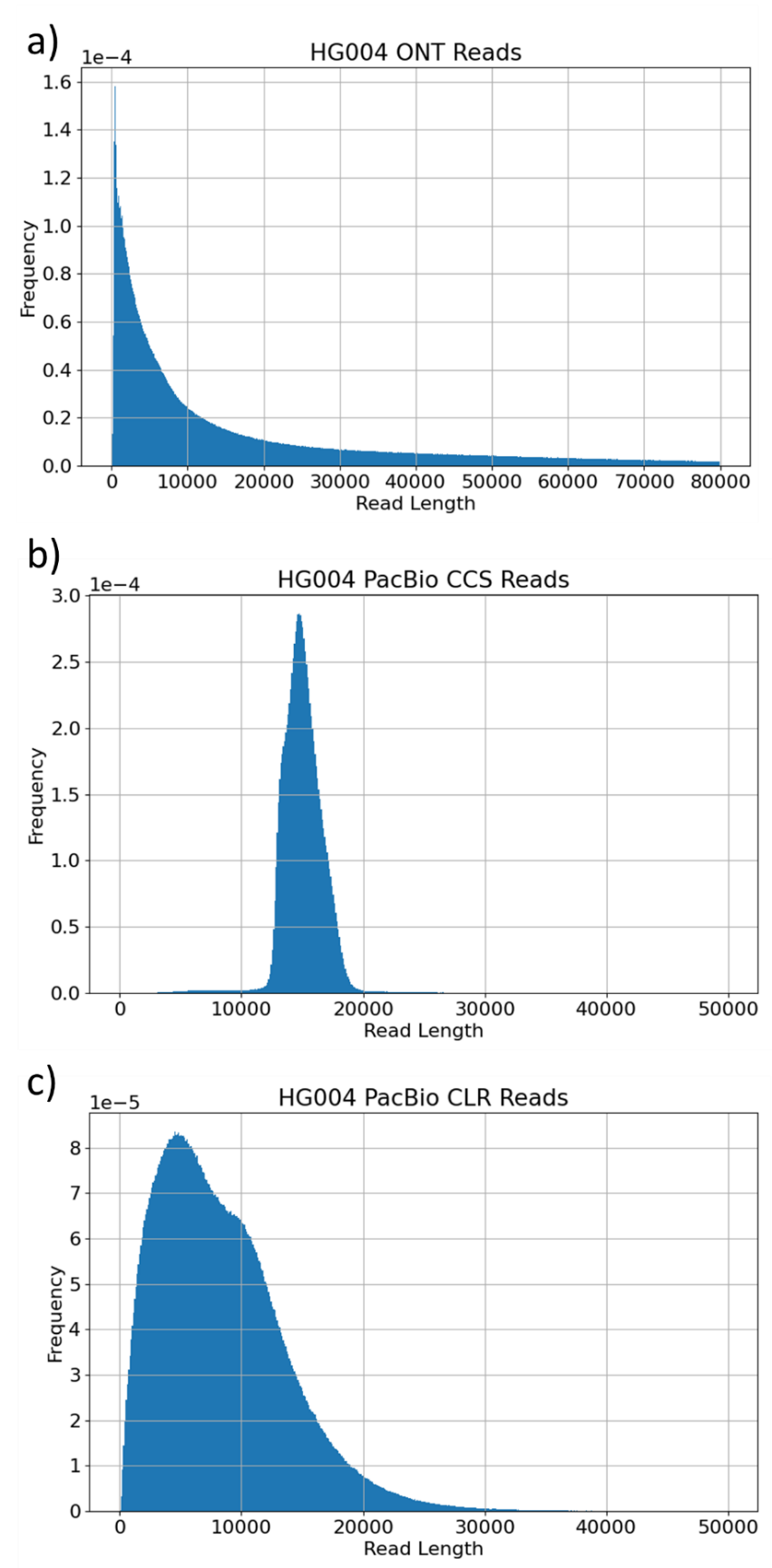


Fig S3. Illustration of number of potentially heterozygous SNP sites chosen in each range for the two methods.


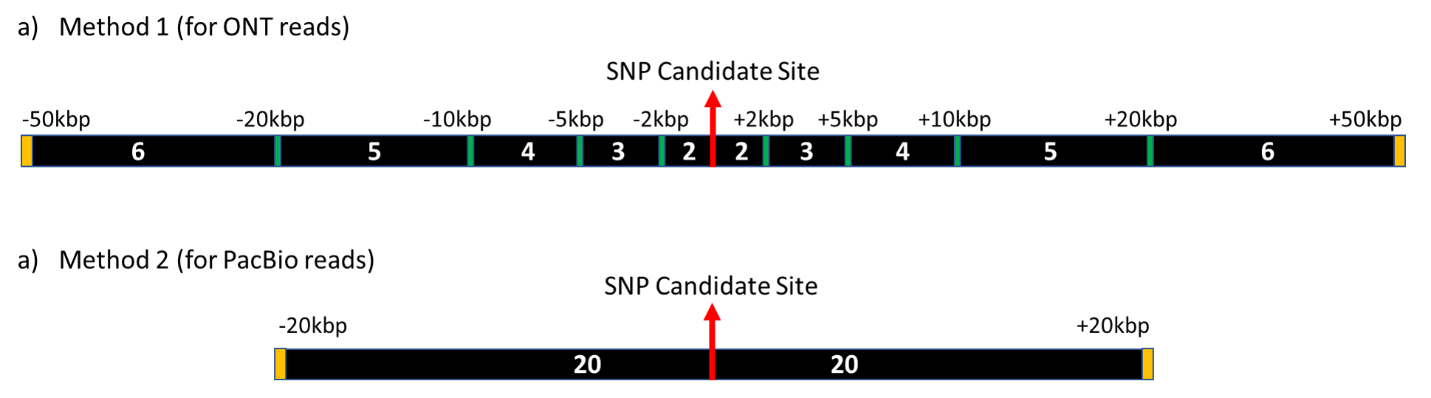


# How to calculate the percentage of high-confidence regions.

We download high-confidence regions for HG001-7 from GIAB. For each of them, we calculate the number of bases in high-confidence regions of chromosomes 1-22, and divide it by the total length of chromosomes 1-22 to obtain the percentage of high-confidence regions. According to our calculation, the percentage of high-confidence regions is 81.05% for HG001, 88.44% for HG002, 87.97% for HG003, 87.83% for HG004, 79.67% for HG005, 81.67% for HG006, and 81.59% for HG007. Roughly, high-confidence regions cover 81-88% of human genome. The total length of high-confidence intervals for chr1-22 are calculated using the command below.

Total length of high-confidence regions:
`cat high_confidence.bed | awk ‘{sum+=$3-$2+1}END{print sum}’`

“high_confidence.bed” is the BED file for GIAB high-confidence intervals.

# The command templates and examples to run NanoCaller and RTG-tools

The commands below can be used to reproduce the results generated by NanoCaller.

For ONT datasets:

*`python NanoCaller_WGS.py -bam HG002.nanopore.bam -ref GRCh38.fa -prefix HG002 -mode both
-seq ont -snp_model ONT-HG002_guppy4.2.2_giab-4.2.1 -indel_model HG002_ont_indel -o output**-sample HG002 -cpu 16 -min_allele_freq 0.15 -min_nbr_sites 1 -exclude_bed hg38 -mincov 8
-maxcov 160 -nbr_t '0.4,0.6' -ins_t 0.4 -del_t 0.6 -win_size 10 -small_win_size 4`*

For PacBio CCS datasets:

` *python NanoCaller_WGS.py -bam HG002.CCS.bam -ref GRCh38.fa -prefix HG002 -mode both
-seq pacbio -snp_model CCS-HG002 -indel_model CCS-HG002 -o output -sample HG002 -cpu 16
-min_allele_freq 0.15 -min_nbr_sites 1 -exclude_bed hg38 -mincov 4 -maxcov 160 -nbr_t '0.3,0.7'
-ins_t 0.4 -del_t 0.4 -win_size 10 -small_win_size 4* `

For PacBio CLR datasets:

` *python NanoCaller_WGS.py -bam HG002.CLR.bam -ref GRCh38.fa -prefix HG002
-mode snps_unphased -seq pacbio -snp_model CLR-HG002 -o output -sample HG002 -cpu 16
-min_allele_freq 0.15 -min_nbr_sites 1 -exclude_bed hg38 -mincov 4 -maxcov 160 -nbr_t '0.3,0.6'*`

Users can replace the reference file (specified by “-ref”), and input BAM file (specify by “-bam”) for different datasets with snp or indel calling or both (specified by “-mode”). The well-trained models for SNP and indel calling can be specified by “-snp_model” and “-indel_model” parameters.

The command template below is used to evaluate predicted VCF against benchmark variant sets.

*`rtg vcfeval -b benchmark.vcf.gz -c variant_calls.vcf.gz -t GRCh38.sdf -e evaluation_region.bed -Z -f ‘QUAL’ -o performance_statistics`*

Users can replace the reference .sdf folder specified by ‘-t’, benchmark VCF file specified by ‘-b’, output VCF file by NanoCaller or other tools specified by ‘-c’, and evaluation regions can be specified by *‘-e’*.

.sdf folder for a reference genome using the following command:

` rtg format -f fasta GRCh38.fa -o GRCh38.sdf`

Creating homopolymer and non-homopolymer evaluation regions for indels.

First, we download GRCh38_AllHomopolymers_gt6bp_imperfectgt10bp_slop5.bed and GRCh38_SimpleRepeat_homopolymer_4to6_slop5.bed BED files from GIAB genome stratification V2.0 as described on ‘Additional file 1’ Page 5.

Commands to create homopolymer regions:

`cat GRCh38_AllHomopolymers_gt6bp_imperfectgt10bp_slop5.bed GRCh38_SimpleRepeat_homopolymer_4to6_slop5.bed |bedtools sort > all_homopolymers.bed`

Commands to intersect homopolymer regions with high-confidence regions:

`bedtools intersect -a high_confidence.bed -b all_homopolymers.bed > high_confidence_homopolymers.bed`

Commands to remove homopolymer regions from high-confidence regions:

`bedtools subtract -a high_confidence.bed -b GRCh38_SimpleRepeat_homopolymer_4to6_slop5.bed| bedtools subtract -a - -b GRCh38_AllHomopolymers_gt6bp_imperfectgt10bp_slop5.bed > high_confidence_minus_homopolymer_repeats.bed`

# Size distribution of indels in GIAB benchmark variant sets.

Table S24. Size distribution of indels in high-confidence intervals of GIAB v4.2.1 benchmarks for HG002-4. Each column is for indels in a specific length range.

|  | 2-10bp | 11-20bp | 21-30bp | 31-40bp | 41-50bp | >50bp |
| --- | --- | --- | --- | --- | --- | --- |
| HG002 | 494630 | 23400 | 5382 | 1479 | 559 | 19 |
| HG003 | 477163 | 21169 | 4573 | 1153 | 439 | 3 |
| HG004 | 483180 | 21289 | 4485 | 1143 | 422 | 0 |

Statistics and performance with old NanoCaller model and datasets.

In Table S26 and Fig S4-Fig S5 below, three NanoCaller SNP models are evaluated: NanoCaller1 (trained on HG001 ONT reads basecalled with Guppy2.3.8), NanoCaller2 (trained on HG002 ONT reads basecalled with Guppy2.3.4), and NanoCaller3 (trained on HG003 PacBio CLR reads). Two NanoCaller indel models are also tested: NanoCaller1 (trained on HG001 ONT reads basecalled with Guppy2.3.8) and NanoCaller3 (trained on HG001 PacBio CCS reads (11kb library size)). Testing datasets include: ONT HG001 reads basecalled by Guppy 2.3.8, ONT HG002-4 reads from precisionFDA challenge basecalled by Guppy 3.6, HX1 reads basecalled with Albacore, HG001 CCS (11kb library size) reads obtained GIAB’s database, and HG002-4 CCS (15kb library size) reads downloaded from precisionFDA truth challenge V2.

Table S25. Statistics of benchmark variants in chromosomes 1-22 of each genome aligned to the GRCh38 reference genome. Four genomes with GIAB benchmark variant calls (with v3.3.2 for HG001 and v4.2 for HG002-4), and statistics within the high confidence regions are also given. For HX1, high confidence regions are created by removing GIAB low complexity regions from the GRCh38 reference genome.

|  | **Whole genome** | | **High confidence region** | | | | **Non homo**  **-polymer**  **region** |
| --- | --- | --- | --- | --- | --- | --- | --- |
| **Genome** | **SNPs** | **Indels** | **SNPs** | **Indels** | **Total Length** | **% of genome** | **Indels** |
| HG001 | 3,004,071 | 516,524 | 2,961,527 | 483,630 | 2,329,784,734 | 81.03 | 180,844 |
| HG002 | 3,459,843 | 587,978 | 3,365,334 | 525,466 | 2,542,282,520 | 88.43 | 210,354 |
| HG003 | 3,430,611 | 569,180 | 3,331,481 | 504,910 | 2,530,414,070 | 88.01 | 199,530 |
| HG004 | 3,454,689 | 576,301 | 3,355,595 | 511,524 | 2,530,026,864 | 88.00 | 201,110 |
| HX1 | 3,282,242 | 687,501 | 2,788,450 | 176,587 | 2,356,619,870 | 75.77 | -- |

Table S26. Performances (Precision/Recall/F1 percentages) of SNP and indel predictions by NanoCaller1, NanoCaller2 and NanoCaller3 on ONT and PacBio (CCS and CLR) data and on difficult-to-map genomic regions along with the performance of existing variant callers. These evaluation is based on v3.3.2 benchmark variants for HG001 and v4.2 benchmark variants for the Ashkenazim trio (HG002, HG003, HG004).

| **Pred** |  | HG001 | HG002 | HG003 | HG004 | HX1 |
| --- | --- | --- | --- | --- | --- | --- |
| For SNPs on  ONT data | NanoCaller1 | 97.29/93.59/95.40 | 98.00/97.99/97.99 | 99.02/98.73/98.88 | 98.85/98.69/98.77 | 90.60/89.96/90.28 |
|  | NanoCaller2 | 96.64/93.21/94.89 | 97.90/98.03/97.97 | 99.16/98.61/98.88 | 99.03/98.61/98.82 | 92.10/89.80/90.94 |
|  | Medaka | 98.29/96.13/97.20 | 98.38/98.66/98.52 | 99.01/99.05/99.03 | 99.07/99.00/99.03 | 97.24/95.16/96.19 |
|  | Clair | 98.79/94.39/96.54 | 98.24/97.28/97.76 | 99.05/97.94/98.49 | 99.00/98.11/98.55 | 96.38/93.66/95.00 |
|  | Longshot | 98.62/95.66/97.12 | 98.93/97.09/98.00 | 98.93/96.83/97.87 | 98.96/96.75/97.84 | 95.40/93.81/94.60 |
|  | WhatsHap |  | 96.33/84.08/89.79 | 97.96/88.34/92.90 | 97.65/80.56/88.28 |  |
| For SNPs on ONT data in difficult regions | Total SNPs |  | 628,157 | 611,270 | 619,719 |  |
|  | NanoCaller1 |  | 96.16/95.04/95.60 | 97.30/96.27/96.78 | 97.23/96.14/96.68 |  |
|  | Medaka |  | 94.74/95.48/95.11 | 96.14/96.54/96.34 | 96.41/96.12/96.27 |  |
|  | Clair |  | 94.68/95.33/95.01 | 96.47/96.11/96.29 | 96.23/96.22/96.22 |  |
|  | Longshot |  | 97.48/91.12/94.19 | 96.84/92.34/94.53 | 96.87/92.06/94.40 |  |
| For indels on ONT data | NanoCaller1 | 49.23/58.09/53.30 | 82.22/77.40/79.74 | 79.22/83.34/81.23 | 77.12/82.49/79.71 |  |
|  | NanoCaller2 | 48.69/56.86/52.46 | 82.45/77.05/79.66 | 79.27/83.06/81.12 | 77.22/82.35/79.70 |  |
|  | Medaka | 75.45/57.09/65.00 | 82.99/75.10/78.85 | 86.47/82.37/84.37 | 85.00/80.38/82.63 |  |
|  | Clair | 81.02/49.75/61.65 | 85.88/58.24/69.41 | 86.00/65.30/74.23 | 85.33/64.70/73.60 |  |
| For all variants on ONT data in MHC | Ensemble |  | 98.30/95.34/96.79 | 98.30/96.56/97.42 | 97.94/96.12/97.02 |  |
|  | NanoCaller1 |  | 97.20/95.22/96.20 | 97.78/96.21/96.99 | 97.48/96.28/96.87 |  |
|  | Medaka |  | 97.19/92.99/95.05 | 98.06/97.62/97.84 | 96.16/89.20/92.55 |  |
|  | Clair |  | 96.92/94.18/95.53 | 97.51/95.73/96.61 | 97.70/95.46/96.57 |  |
| For SNPs on PacBio CCS data | NanoCaller1 | 96.97/99.37/98.15 | 99.31/99.60/99.45 | 99.36/99.63/99.49 | 99.37/99.63/99.50 |  |
|  | NanoCaller3 | 96.69/99.36/98.01 | 99.16/99.56/99.36 | 99.22/99.58/99.40 | 99.23/99.59/99.41 |  |
|  | Clair | 99.59/99.77/99.68 | 99.83/99.71/99.77 | 99.82/99.70/99.76 | 99.80/99.68/99.74 |  |
|  | Longshot | 99.73/99.04/99.39 | 99.81/98.26/99.03 | 99.82/98.25/99.03 | 99.82/98.22/99.02 |  |
|  | WhatsHap | 98.86/99.63/99.24 | 99.73/98.52/99.12 | 99.74/99.59/99.66 | 99.75/99.57/99.66 |  |
|  | DeepVariant | 99.75/99.86/99.81 | 99.9/99.81/99.85 | 99.87/99.8/99.83 | 99.89/99.8/99.84 |  |
| For indels on PacBio CCS data | NanoCaller1 | 94.39/93.78/94.09 | 94.74/94.07/94.40 | 92.42/94.67/93.53 | 92.02/94.76/93.37 |  |
|  | NanoCaller3 | 94.33/93.71/94.02 | 94.65/93.96/94.30 | 92.32/94.55/93.42 | 91.90/94.66/93.26 |  |
|  | Clair | 96.14/94.60/95.36 | 94.98/95.04/95.01 | 96.64/96.17/96.40 | 96.57/96.06/96.31 |  |
|  | DeepVariant | 97.68/97.52/97.60 | 98.14/98.39/98.26 | 98.31/98.71/98.51 | 98.28/98.66/98.47 |  |
| For SNPs on PacBio CLR data | NanoCaller1 | 97.85/87.08/92.15 | 98.64/96.58/97.60 | 94.48/91.83/93.14 | 93.26/91.03/92.13 |  |
|  | NanoCaller3 | 97.54/85.66/91.21 | 98.43/94.78/96.57 | 93.35/88.77/91.00 | 92.10/88.01/90.01 |  |
|  | Clair | 99.10/92.77/95.83 | 98.22/98.54/98.38 | 95.69/94.11/94.89 | 95.03/93.28/94.15 |  |
|  | Longshot | 99.50/94.26/96.81 | 99.02/97.80/98.41 | 99.4/89.79/94.35 | 99.38/87.87/93.27 |  |

Fig S4. Performance of NanoCaller and state-of-the-art variant callers on five whole-genome Oxford Nanopore sequencing data sets. The performance of SNP predictions on ONT reads: a) precision, b) recall, c) F1 score. d) The performance of SNP predictions on HG002 (ONT), HG003 (ONT) and HG004 (ONT) in difficult-to-map genomic regions. The performance of variant predictions on HG002 (ONT), HG003 (ONT) and HG004 (ONT) in Major Histocompatibility Complex regions: e) SNPs, f) overall variants. The performance of indel predictions on ONT reads in non-homopolymer regions: g) precision, h) recall, i) F1 score. For HX1, the variants, which were called on high-coverage short-read data are used as benchmark with complement of difficult-to-map genomic regions used in d) as high-confidence regions. Benchmark variants v3.3.2 for HG001 and v4.2 for the Ashkenazim trio (HG002, HG003, HG004) are used for evaluation.


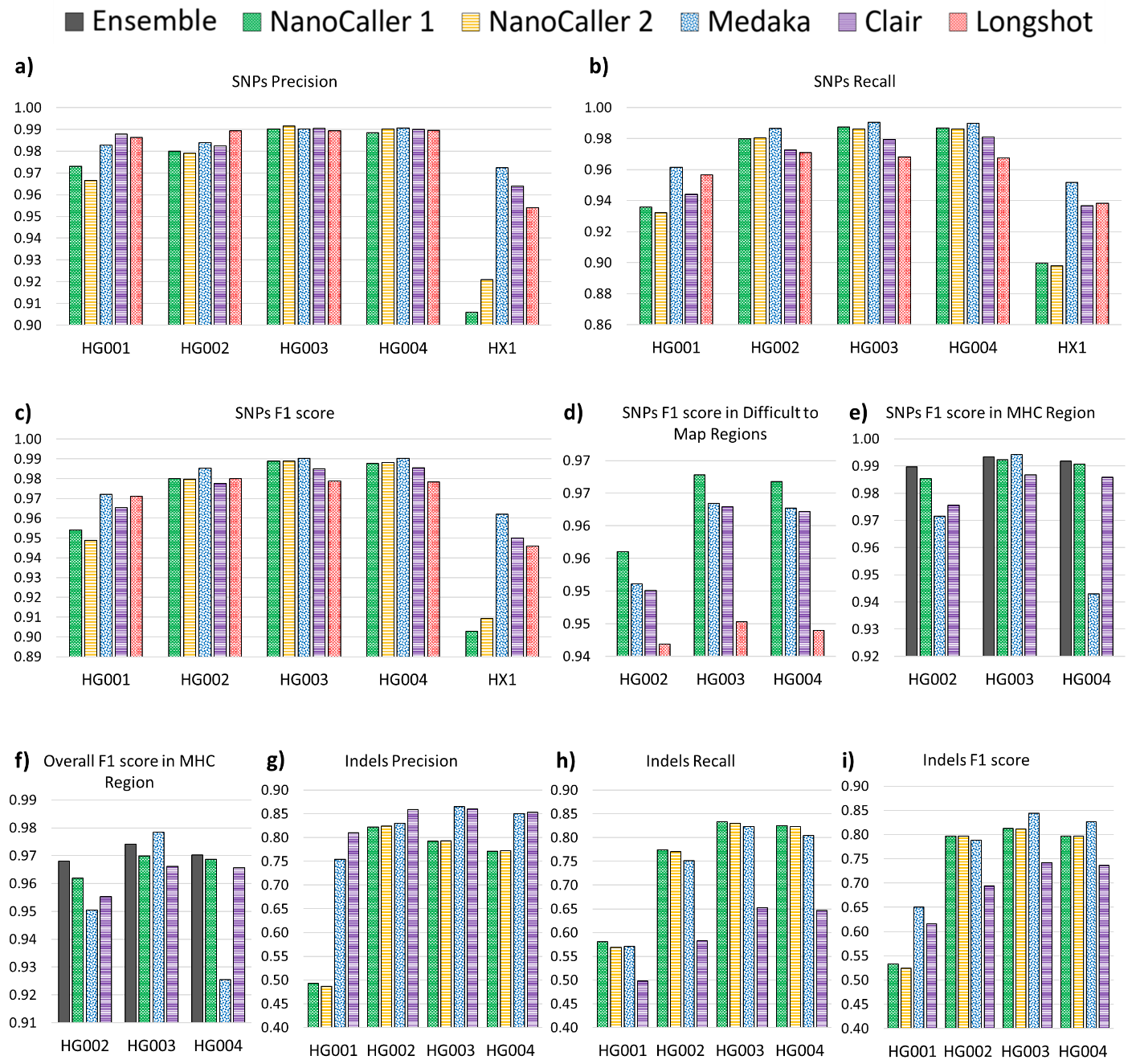


Fig S5. Performance of NanoCaller and state-of-the-art variant callers on PacBio sequencing data sets. The performance of SNP predictions on PacBio CCS reads: a) precision, b) recall, c) F1 score. The performance of indel predictions on PacBio CCS reads: d) precision, e) recall, f) F1 score. The performance of SNP predictions on PacBio CLR reads: g) precision, h) recall, i) F1 score. Benchmark variants v3.3.2 for HG001 and v4.2 for the Ashkenazim trio (HG002, HG003, HG004) are used for evaluation.


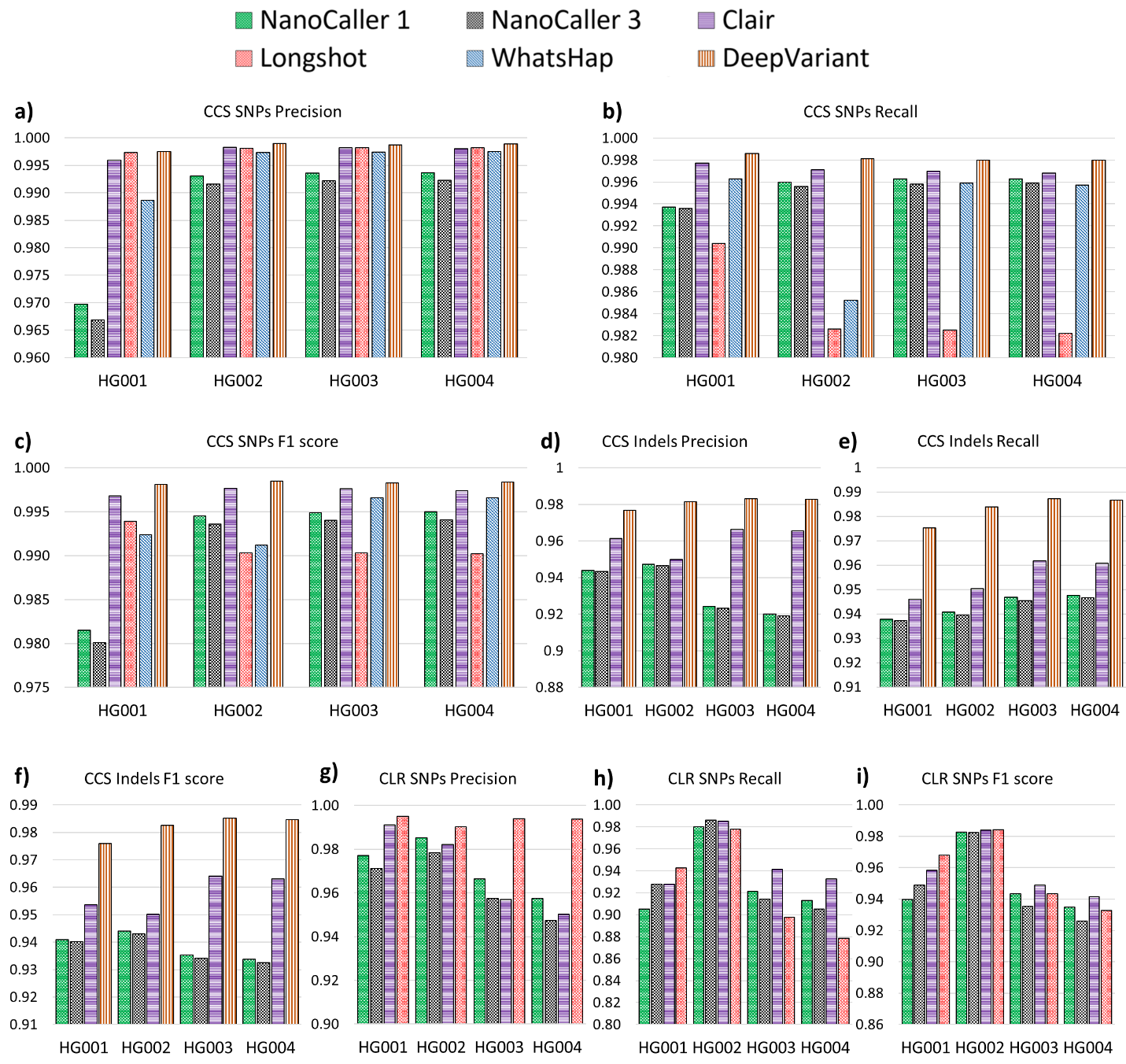


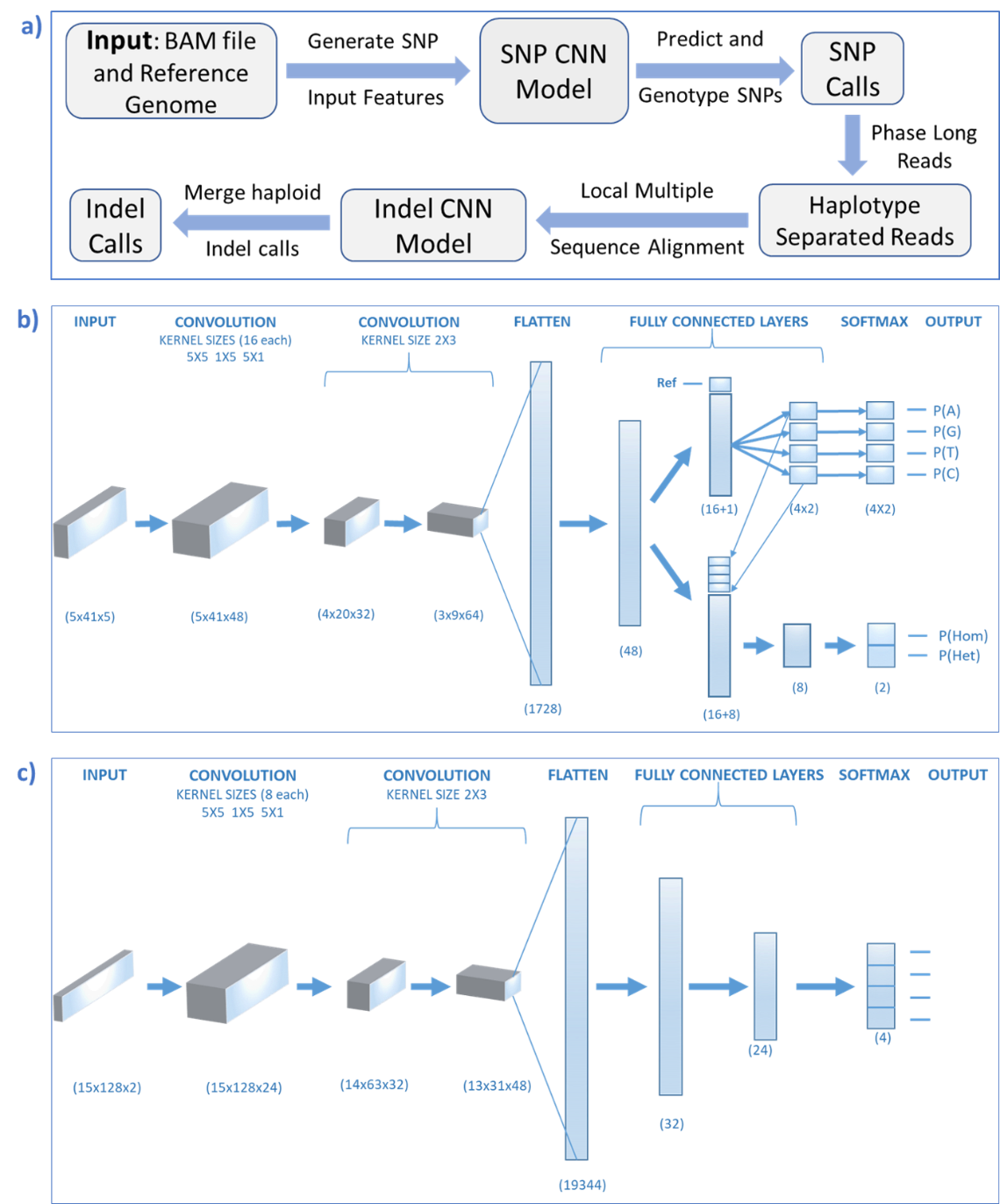
Fig S6. The deep-learning framework for SNP and indel calling. a) The overall workflow of NanoCaller. b) An illustration of the convolutional neural network model for SNP calling; c) An illustration of the convolutional neural network model for indel calling. In both models, first convolutional layer uses 3 kernels of sizes 1x5, 5x1 and 5x5, whereas the second and third convolutional layers use kernels of size 2x3. Output of third convolutional layer is flattened and fed to a fully connected layer followed by a hidden layer with 48 nodes with a 50% dropped rate. In b), output of the hidden layer is split into two independent pathways: one for calculating probabilities of each base and the other for calculating zygosity probabilities. Zygosity probability is only used in the training process. In c), output of first fully connected layer is fed into two fully connected hidden layers to produce probabilities of four possible cases of zygosities.


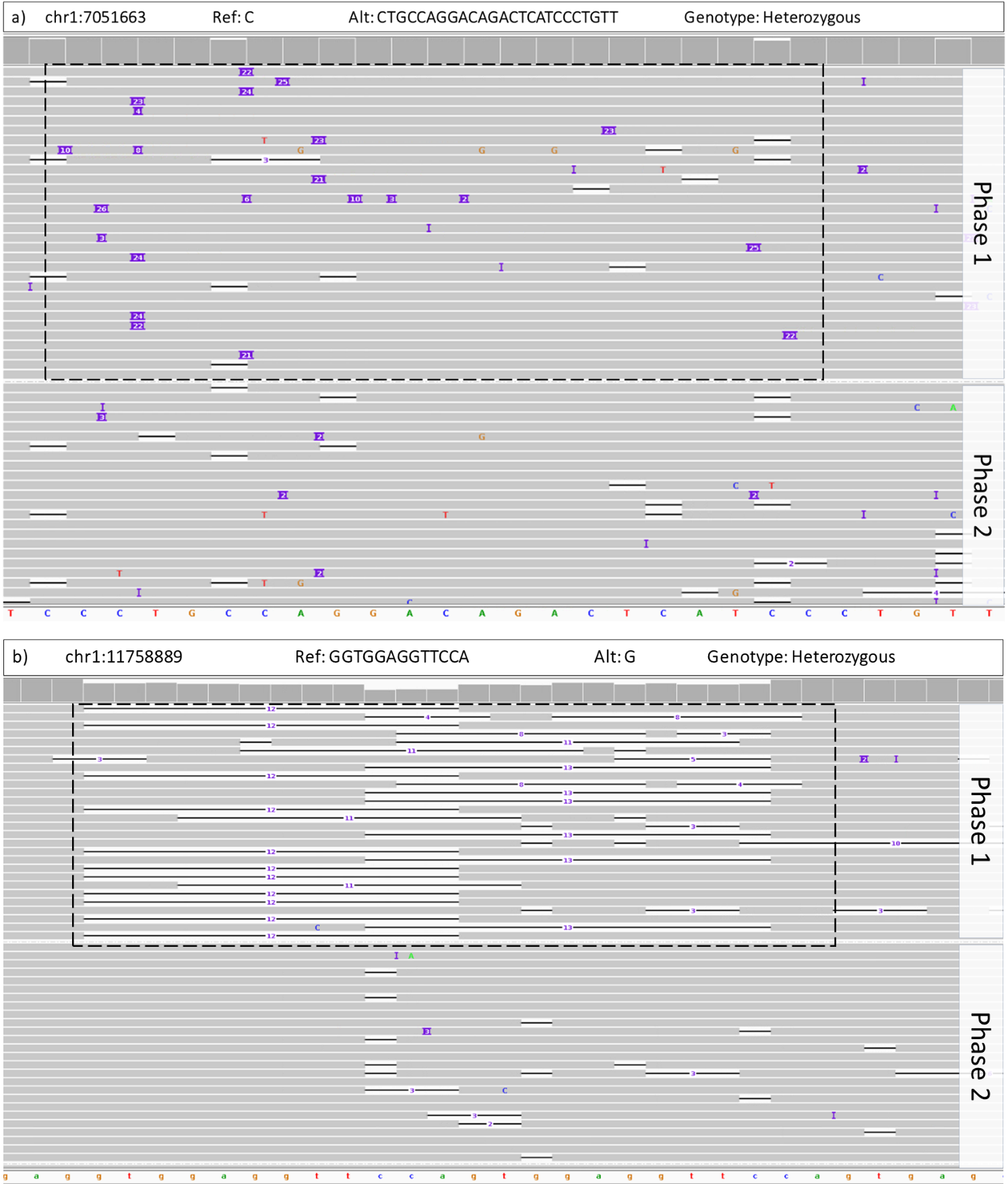


Fig S7. Two indels from HG002 GIAB benchmark v4.2.1 identified correctly by NanoCaller but missed by Clair and Medaka. Both indels lie in tandem repeats, therefore with high discordance among ONT reads for the locus of the indel (shown in black rectangle). NanoCaller uses a sliding window to detect such indels hat do not have high allele frequency at any single reference genome locus. a) An insertion; b) An deletion.
