# Supplementary material for: NanoCaller for accurate detection of SNPs and indels in difficult-to-map regions from long-read sequencing by haplotype-aware deep neural networks
Source: Genome Biol. 2021 Sep 6;22:261. doi: 10.1186/s13059-021-02472-2 (PMC8419925; doi:10.1186/s13059-021-02472-2)
Supplement: Supplementary file 4 — Additional file 4. : Review history. [file 13059_2021_2472_MOESM4_ESM.docx]

**1^st^ round**

**Reviewer 1**

In this manuscript, the authors proposed a new approach for accurate detection of SNPs and indels from long-read sequencing data.

main problem:

1) For nanopore data, does nanocaller consider homopolymer, such as AAAAAA, or quasi-homopolymer, such as AAATAAAA? Because the signal of nanopore sequencing in this area is unstable, the length of the homopolymer or the accuracy of sequencing are reduced. If nanocaller has a special plan to deal with this problem, please list it in the text or supplementary materials. If not, please do a comparison test with other methods on the above data;

2) At present, many long-read SNP calling methods on the market are based on machine learning. One of my concerns is that even if the training data/test data of nanocaller is consistent with competing products, how to ensure that when we are in a different data set (assuming we use human data), or a different species (for example: ecoli or other bacteria), can SNP calling ensure consistent accuracy?

3) The result that Nanocaller makes me shine is on difficult-to-map and MHC region. However, the article does not seem to emphasize why nanocaller has an advantage in this part of the data compared to other competing products.

4) Although it performs well on difficult data, nanocaller does not seem to exceed the current competitors on the market on ordinary data sets, especially on the HX1 data set (see Figure 4). I did not find any relevant result analysis in the text, please add.

5) A similar problem, Figure 5 shows that the performance of nanocaller does not significantly exceed deepvariant. Does this mean that the advantage of nanocaller on pacbio is not significant? Another interesting question is that by definition, nanocaller3 is trained on pacbio data, and nanocaller1&2 is trained on nanopore data. However, from the results in Figure 5, nanocaller3 does not seem to exceed nanopore1 significantly. Please explain why.

Secondary issues:

1) Figure 1 hopes to make a better supplement, to draw the entire flow chart more clearly, and hope it can be more beautiful.

2) In Figure 2, why is the error rate of the first few positions of the reads in the Ra area so high? Will this cause unnecessary misunderstandings to readers?

3) The 26th sentence on the first page of the Introduction, "for example, only ~80% of genomic regions are marked as 'high-confidence region", please give the quotation.

4) The 47th sentence on the first page of Introduction, "However, the per-base accuracy of long reads is much lower with raw base calling errors of 3-15%", is the 15% error rate here a bit high? Because the current nanopore protein R10.3 can already make raw read accuracy reach 95%.

5) The 48th sentence on the second page of Introduction, "there may be room in further improving these approaches especially for difficult-to-map regions", I did not see the specific explanation of nanocaller to solve these difficulties, please add.

**Reviewer 2**

The paper describes NanoCaller, a deep-learning small variant caller for long-read sequence data, including comparative performance of this tool in the recent PrecisionFDA2 Truth Challenge. This is a timely publication, as interest is growing in long-read sequencing, and I am pleased to see it.

Regrettably, I have not had the time in the two weeks of this review to test NanoCaller directly, although I hope to do so soon.

Please consider the following questions:

- Are the methods appropriate to the aims of the study, are they well described, and are necessary controls included?

This is both a presentation of the method itself (variant caller software) and a description of the validation of that software. The method seems appropriate and is described in great detail. Because the validation was performed as part of a public, independently-evaluated competition, this is about as appropriate as you can get for a small variant caller. Controls used were PacBio and Oxford Nanopore sequence data generated from standard cell lines, which have gold-standard Genome in a Bottle small variant data available. These are the most appropriate controls possible.

- Are the conclusions adequately supported by the data shown?

Yes.

- Are sufficient details provided to allow replication and comparison with related analyses that may have been performed?

In general, yes, although I have some comments below.

- Is the method likely to be of broad utility? Is any software component easy to install and use? Please indicate briefly the novel features and/or advantages of the method, and/or please reference the relevant publications and which methods, if any, it should be compared with.

Absolutely -- given the accuracy of the method, there is a high chance that it will become one of the standard small variant callers for PacBio and ONT data.

- Is the paper of broad interest to others in the field, or of outstanding interest to a broad audience of biologists?

The paper is somewhat light on biology (apart from some interesting results validating novel variants in difficult-to-sequence regions). But it is definitely of great interest to the more clinical side of the community interested in small variant calling.

Some specific comments that I would like to have addressed:

1. I would like to see more detail about the code used for performance measurement. Matching indel calls (and even SNV calls) requires some level of normalisation, as different callers may choose different representations of the same variant. Our go-to has been rtg-tools, but I would like to know what was used for the comparisons.

2. I couldn't find details on the exact command/parameters used to run nanocaller for the results presented. Recommended parameters are mentioned on the Github, but it's not clear if that was what was run. Also I expect that the Github will change over time. Please ensure that this information is included either as supplementary information, or in the paper, or in a separate Github repo.

3. Please define in the paper what your size range is for an indel. 1-50bp is fairly common, and I suspect is the definition used by GIAB, but it's good to be explicit. With long-read sequencing, the line between small indels and structural variant indels is blurring.

4. Further on this topic, could you please comment in the paper on how NanoCaller performs on indels based on their size? How large of a variant can it call? Would it work with the recently-published GIAB SV calls for hg0002?

5. The ONT data used in this publication was basecalled using Guppy 2.3.x. Guppy has since undergone two major upgrades to its accuracy, in 3.6.0 and again in 4.4.0 with the introduction of the Rerio/Bonito models. While it would be too much to ask for all of the analysis in this paper to be re-run (especially since it was part of the PrecisionFDA challenge), I would like to see how NanoCaller performs on the newer, more-accurate basecalls, at least for one data set. ONT provide a public data set for hg0002, which I believe could be used for this purpose:

https://nanoporetech.github.io/ont-open-datasets/gm24385_2020.09/

5. The GIAB calls make for excellent training data, but raise the issue of maintaining a train/test split when evaluating software. To this end you have split your models into NanoCaller1 and NanoCaller2 for Oxford Nanopore. The authors of clair did something similar, but they also trained a model on all of the GIAB data available, for general use on non-GIAB data. Could you please explain why you have not done this?

6. Small variant calling has two major use cases in human health: resolving hereditary/germline traits, and calling driver mutations in cancer. The GIAB data makes for an excellent training and testing set for the first case. However, there is also a need for small variant callers for long-read sequence data from tumours. This data poses challenges for variant calling, including clonal heterogeneity and variable tumour content in the samples sequenced. Could you please comment in the paper on the appropriateness (or not) of NanoCaller for tumour small variant calling?

**Authors’ response**

Reviewer #1: In this manuscript, the authors proposed a new approach for accurate detection of SNPs and indels from long-read sequencing data.
main problem:
1) For nanopore data, does nanocaller consider homopolymer, such as AAAAAA, or quasi-homopolymer, such as AAATAAAA? Because the signal of nanopore sequencing in this area is unstable, the length of the homopolymer or the accuracy of sequencing are reduced. If nanocaller has a special plan to deal with this problem, please list it in the text or supplementary materials. If not, please do a comparison test with other methods on the above data;
**Authors’ Response**: Thanks for this comment. We agree with you that (quasi-)homopolymer regions have reduced accuracy of basecalling and variant calling. In NanoCaller, we use the same strategy to make SNP/indel calls for homopolymer regions and other regions (the detail definition can be found in Pages 5 & 6 of “Supplementary Materials 1”).

To address your comment, we test NanoCaller/Medaka/Clair on homopolymer regions defined by GIAB in their genome stratification release ([*https://ftp-trace.ncbi.nlm.nih.gov/ReferenceSamples/giab/release/genome-stratifications/v2.0/GRCh38/LowComplexity/v2.0-GRCh38-LowComplexity-README.txt*](https://ftp-trace.ncbi.nlm.nih.gov/ReferenceSamples/giab/release/genome-stratifications/v2.0/GRCh38/LowComplexity/v2.0-GRCh38-LowComplexity-README.txt) *where, similar to your definition, homopolymer regions are regions with perfect homopolymers (such as AAAAAA) with >=4bp and imperfect homopolymers (such as AAAATAAAAAA) with “a single base was repeated >10bp except for a 1bp interruption by a different base”.*). With the evaluation with homopolymer regions (as shown in Table S31 in “Supplementary Materials 2”), we find that NanoCaller indel F1-scores exceed Clair and Medaka’s performance on 6 of 7 tested genomes, and on the other genome, NanoCaller has better performance than Clair. This suggests that NanoCaller generally outperforms other methods to call variants from homopolymer regions, although there is no specialized process of indel calling for homopolymer regions.

2) At present, many long-read SNP calling methods on the market are based on machine learning. One of my concerns is that even if the training data/test data of nanocaller is consistent with competing products, how to ensure that when we are in a different data set (assuming we use human data), or a different species (for example: ecoli or other bacteria), can SNP calling ensure consistent accuracy?
**Authors’ Response**: This is a good point about the real-world application of NanoCaller. To make NanoCaller useful in other datasets, we mainly test NanoCaller under cross-genome independent testing: *that is, NanoCaller is trained on a genome of an individual, and then tested on several other genomes for different individuals (usually sequenced in different centers or batches)*. This is a widely-used blind testing strategy to ensure that well-trained models are not specific to training datasets. According to cross-genome blind testing (as shown in Table S30/S37/S39 in ‘Supplementary Materials 2’, and Page 7/Table 2/Figures 3&4 of main manuscript) on 8 different human genomes, we find that NanoCaller always has F1 score >0.98 on ONT data of 8 human genomes, and >0.99 on Pacbio CCS data of 4 genomes, although NanoCaller was trained on HG002 only. Meanwhile, we participated in PrecisionFDA truth challenge V2, and the benchmark variant sets during the challenge is not disclosed prior to the challenge. During this challenge, NanoCaller also has the consistent performance of variant prediction. Thus, we are confident that NanoCaller can generate consistent accuracy on the different human sequencing data generated by PacBio and Nanopore sequencing. We will be happy to test NanoCaller on long-read data for other non-diploid species (such as E. coli and other bacteria), once the corresponding benchmark “truth” variant sets are available.

3) The result that Nanocaller makes me shine is on difficult-to-map and MHC region. However, the article does not seem to emphasize why nanocaller has an advantage in this part of the data compared to other competing products.
**Authors’ Response**: Thank you very much for this comment, and we are glad that NanoCaller works well on difficulty-to-map regions. Per the comment about why NanoCaller has this advantage, we split difficult-to-map regions into different subgroups according to their length: 0-10kbp, 10-100kbp, 100-500kbp, and >500kbp, and test NanoCaller, Medaka, Clair and Longshot on HG002, HG003 and HG004. We find that when the length of interval increases, the improvement of NanoCaller over other methods becomes more obvious: for example on HG004, NanoCaller’s F1-score is 0.02 higher than Longshot for 0-10kbp subgroup, while NanoCaller’s F1-score is 0.1793 higher than Longshot for >500kbp. More comparison with Medaka and Clair on more genomes can be found in Table S41 in ‘Supplementary Materials 2’. We add this discussion in Page 9 of main manuscript.

Meanwhile, we train a SNP model that creates SNP input features using only the 20 immediately adjacent bases on each side of the candidate site. With the same training and testing process, we find that NanoCaller with long-range information generally has 0.5-3% improvement (as shown in Table R1 below). Both evaluations demonstrate that long-range haplotype information from neighboring (several kb) downstream and upstream region helps NanoCaller prediction especially on difficult-to-map regions.

**Table R1. Comparison of SNP F1-scores for both types of models on ONT HG002-4 Guppy4.2.2 reads evaluated against v4.2.1 benchmark variants, in whole genome, “all difficult-to-map” regions and MHC.**

|  | **Genome** | **NanoCaller Long-range information Model** | **Adjacent Bases Model** |
| --- | --- | --- | --- |
| Whole Genome | HG002 | 0.9866 | 0.9751 |
|  | HG003 | 0.9910 | 0.9851 |
|  | HG004 | 0.9912 | 0.9849 |
| All 'difficult-to-map' regions | HG002 | 0.9618 | 0.9454 |
|  | HG003 | 0.9692 | 0.9598 |
|  | HG004 | 0.9692 | 0.9577 |
| MHC | HG002 | 0.9888 | 0.9635 |
|  | HG003 | 0.9920 | 0.9832 |
|  | HG004 | 0.9929 | 0.9794 |

4) Although it performs well on difficult data, nanocaller does not seem to exceed the current competitors on the market on ordinary data sets, especially on the HX1 data set (see Figure 4). I did not find any relevant result analysis in the text, please add.
**Authors’ Response**: Thanks for this comment. To address this comment and other comments raised by other reviewers regarding out-of-date basecalling, we re-train NanoCaller on latest Nanopore data, and test NanoCaller and other variant callers on latest Nanopore dataset of HG001-7 for whole genome SNP evaluation. On one hand, we find that NanoCaller has better SNP performance than Medaka, Clair and Longshot on the Ashkenazim trio HG002-4 with more extensive and up to date benchmark variants, and exceeds Clair by significant margins on HG005-7, and performs competitively with other methods on HG001 and HG005-7 (as shown in Table S30 in ‘Supplementary Materials 2’, and Pages 7&8/Table 2/Figures 3&4 of main manuscript).

Additionally, although HX1 was not a benchmarking data set from NIST (as the truth is purely defined by GATK-based call in Illumina data), we re-basecall HX1 with latest guppy version 4.5.2 (previously, HX1 dataset was basecalled using Albacore), and test NanoCaller on the newly basecalled HX1 data. We find that NanoCaller performs better than Clair and Longshot, and competitively Medaka (as shown in Table S30 in ‘Supplementary Materials 2’ and Page 8/Table 2/Figures 3&4 of main manuscript).

5) A similar problem, Figure 5 shows that the performance of nanocaller does not significantly exceed deepvariant. Does this mean that the advantage of nanocaller on pacbio is not significant? Another interesting question is that by definition, nanocaller3 is trained on pacbio data, and nanocaller1&2 is trained on nanopore data. However, from the results in Figure 5, nanocaller3 does not seem to exceed nanopore1 significantly. Please explain why.
**Authors’ Response**: Thanks for this comment. (1) NanoCaller is designed to tolerate high error rate. In our testing, we find that NanoCaller is able to achieve competitive performance against deepvariant on CCS reads (with lower error rate) but better on other data with high error rates (PacBio CLR reads and ONT reads). (2) The situation of the second question is due to that NanoCaller3 was trained on low coverage (28x) Pacbio CLR data for HG003 and NanoCaller3 trained on CLR reads was tested on CCS reads (*Please note that Pacbio CCS reads and CLR reads/Nanopore reads have different error profiles*). To address this comment, on one hand, we re-train a new model on HG002 Pacbio CLR data with ~60X coverage. We find that this new model performs better than new ONT models on PacBio continuous long reads (i.e. CLR or raw reads). Please refer to Page 11 of main manuscript and in Table S39 in ‘Supplementary Materials 2’ for details. On the other hand, we also train new NanoCaller models on CCS reads and test them on PacBio CCS reads together with the models trained on Nanopore data, and we also find that the CCS models performs slightly better than ONT models on PacBio CCS reads (see Page 11 of main manuscript and in Table S37 in ‘Supplementary Materials 2’).

Secondary issues:
1) Figure 1 hopes to make a better supplement, to draw the entire flow chart more clearly, and hope it can be more beautiful.
**Authors’ Response**: Thank you for your comments. We improved original Figure 1 and moved it to the supplementary materials. Please refer to Page 33 Figure S6 of ‘Supplementary Materials 1’ for detail.

2) In Figure 2, why is the error rate of the first few positions of the reads in the Ra area so high? Will this cause unnecessary misunderstandings to readers?
**Authors’ Response**: Thank you for pointing this confusing part out. To address your comment, we have improved this figure, and make the process and the explanation clearer. Please refer to Page 34 ( Figure 1 *i.e.*, old Figure 2) of main manuscript for detail. Meanwhile, please note that (1) the first few positions in panel (b) are not directly adjacent but can be hundreds of bp away from each other. These positions correspond to SNPs estimated over long-range and their reference alleles. (2) The error rate compared against the reference genome (i.e. difference between reference and bases in each column) might be higher for Ra, or Rc or Rt, since they contain long-range potential SNPs. If two SNPs co-occur with several supporting long reads, they have higher probability to be true SNPs; otherwise, the difference of bases within each group Ra, Rt or Rc might be due to the sequencing/alignment errors. In NanoCaller, the deep-learning process is designed to learn this. The different error rates suggest true and false SNPs, which we now clarified in the revised figure.

3) The 26th sentence on the first page of the Introduction, "for example, only ~80% of genomic regions are marked as 'high-confidence region", please give the quotation.
**Authors’ Response**: Thank you for this comment. We download high-confidence regions for HG001-7 from the GIAB data set from NIST. Please note that their definition is not based on the GRCh37/GRCh38 reference genome, but based on each variant calling set, so it is a variable number for each reference data set. For each of them, we calculate the number of bases in high-confidence regions of chromosomes 1-22, and divide it by the total length of chromosomes 1-22 to obtain the percentage of high-confidence regions. According to our calculation, the percentage of high-confident regions is 81.05% for HG001, 88.44% for HG002, 87.97% for HG003, 87.83% for HG004, 79.67% for HG005, 81.67% for HG006, and 81.59% for HG007. Roughly, high-confidence regions cover 81-88% of human genome. Please note that the v4.2.1 benchmark high-confidence regions of HG002-4 contains results from released long-read data and thus have higher percentage. We add how to download and calculate this percentage in the ‘Supplementary Materials 1’ Page 26.

4) The 47th sentence on the first page of Introduction, "However, the per-base accuracy of long reads is much lower with raw base calling errors of 3-15%", is the 15% error rate here a bit high? Because the current nanopore protein R10.3 can already make raw read accuracy reach 95%.

**Authors’ Response**: Thanks for pinpointing this out. We agree with you that R10.3 has improved the raw read accuracy. However, the error is still much higher than that of short-read data (we add this discussion in Introduction at Page 3 of main manuscript to make this point clearer according to your comment). Also, please note that many other Nanopore data were generated by other versions of Nanopore flowcells whose error rate is still higher. (In fact, due to technical issues and the low throughput of R10.3 flowcells, as of today, all our own studies and the studies with our collaborators still use R9 rather than R10 to generate data, so there is no widespread community adoption of the R10 cells).

5) The 48th sentence on the second page of Introduction, "there may be room in further improving these approaches especially for difficult-to-map regions", I did not see the specific explanation of nanocaller to solve these difficulties, please add.
**Authors’ Response**: Thank you for this comment. We believe that long-range haplotype information can overcome local errors to improve difficult-to-map regions. As we responded to Comment 3, there are 2 pieces of supporting evidence for this. 1) The difficult-to-map regions are split into different subgroups according to their length, and tested with NanoCaller, Medaka, Clair and LongShot for HG002, HG003 and HG004. We find that when the length of interval increases, the improvement of NanoCaller over other methods becomes larger (as shown in in Table S41 in ‘Supplementary Materials 2’). 2) We train a SNP model that creates SNP input features using only the 20 immediately adjacent bases on each side of the candidate site. With the same training and testing process, we find that NanoCaller with long-range information generally has 0.5-3% improvement (as shown in Table R1 above). Both evaluations demonstrate that long-range haplotype information helps NanoCaller prediction on difficult-to-map regions.

----------------------- REVIEW 2 ---------------------
Reviewer #2: The paper describes NanoCaller, a deep-learning small variant caller for long-read sequence data, including comparative performance of this tool in the recent PrecisionFDA2 Truth Challenge. This is a timely publication, as interest is growing in long-read sequencing, and I am pleased to see it.
**Authors’ Response**: Thank you for your great comments. We strongly agree with you about this.

Regrettably, I have not had the time in the two weeks of this review to test NanoCaller directly, although I hope to do so soon.
**Authors’ Response**: Thank you very much for your review during your busy schedule.

Please consider the following questions:
- Are the methods appropriate to the aims of the study, are they well described, and are necessary controls included?
*This is both a presentation of the method itself (variant caller software) and a description of the validation of that software. The method seems appropriate and is described in great detail. Because the validation was performed as part of a public, independently-evaluated competition, this is about as appropriate as you can get for a small variant caller. Controls used were PacBio and Oxford Nanopore sequence data generated from standard cell lines, which have gold-standard Genome in a Bottle small variant data available. These are the most appropriate controls possible.*
- Are the conclusions adequately supported by the data shown?
*Yes.*
- Are sufficient details provided to allow replication and comparison with related analyses that may have been performed?
*In general, yes, although I have some comments below.*
- Is the method likely to be of broad utility? Is any software component easy to install and use? Please indicate briefly the novel features and/or advantages of the method, and/or please reference the relevant publications and which methods, if any, it should be compared with.
*Absolutely -- given the accuracy of the method, there is a high chance that it will become one of the standard small variant callers for PacBio and ONT data.*
- Is the paper of broad interest to others in the field, or of outstanding interest to a broad audience of biologists?
*The paper is somewhat light on biology (apart from some interesting results validating novel variants in difficult-to-sequence regions). But it is definitely of great interest to the more clinical side of the community interested in small variant calling.*
**Authors’ Response**: Thank you very much for your great comments. We have responded your comments point-by-point below.

Some specific comments that I would like to have addressed:
1. I would like to see more detail about the code used for performance measurement. Matching indel calls (and even SNV calls) requires some level of normalisation, as different callers may choose different representations of the same variant. Our go-to has been rtg-tools, but I would like to know what was used for the comparisons.
**Authors’ Response**: Thank you for this comment. We agree with you that normalization is needed for SNV/indel evaluation. We have thus used rtg-tools for this performance evaluation. To make it explicit for users, we add all the commands we used for performance evaluation in the supplementary materials now. Please refer to Page 27 of the ‘Supplementary Materials 1’ for detail. For your quick reference, the command template is:

*`rtg vcfeval -b benchmark.vcf.gz -c variant_calls.vcf.gz -t GRCh38.sdf -e evaluation_region.bed -Z -f ‘QUAL’ -o performance_statistics`*

Users can replace the reference .sdf folder specified by ‘-t’, the benchmark VCF file specified by ‘-b’, the output VCF file by NanoCaller or other tools specified by ‘-c’, and the evaluation regions can be specified by ‘-e’.

.sdf folder for a reference genome can be generated using the following command:

*` rtg format -f fasta GRCh38.fa -o GRCh38.sdf`*

2. I couldn't find details on the exact command/parameters used to run nanocaller for the results presented. Recommended parameters are mentioned on the Github, but it's not clear if that was what was run. Also I expect that the Github will change over time. Please ensure that this information is included either as supplementary information, or in the paper, or in a separate Github repo.
**Authors’ Response**: Thank you very much for this comment, and we also believe that the commands are critical for users to reproduce the results. We thus added all the commands we run in the supplementary materials. Please refer to Page 26 of the ‘Supplementary Materials 1’ for detail. For your quick reference, the commands are:

For ONT datasets:

*`python NanoCaller_WGS.py -bam HG002.nanopore.bam -ref GRCh38.fa -prefix HG002 -mode both -seq ont -snp_model ONT-HG002_guppy4.2.2_giab-4.2.1	-indel_model HG002_ont_indel	-o output* *-sample HG002	-cpu 16 -min_allele_freq 0.15	-min_nbr_sites 1	-exclude_bed hg38	-mincov 8 -maxcov 160	-nbr_t '0.4,0.6'	-ins_t 0.4	-del_t 0.6	-win_size 10	-small_win_size 4`*

For PacBio CCS datasets:

*` python NanoCaller_WGS.py -bam HG002.CCS.bam	-ref GRCh38.fa	-prefix HG002	-mode both -seq pacbio	-snp_model CCS-HG002	-indel_model CCS-HG002	-o output -sample HG002	-cpu 16 -min_allele_freq 0.15	-min_nbr_sites 1	-exclude_bed hg38	-mincov 4	-maxcov 160	-nbr_t '0.3,0.7' -ins_t 0.4 -del_t 0.4 -win_size 10 -small_win_size 4 `*

For PacBio CLR datasets:

*` python NanoCaller_WGS.py -bam HG002.CLR.bam	-ref GRCh38.fa	-prefix HG002
-mode snps_unphased	-seq pacbio	-snp_model CLR-HG002	-o output	-sample HG002	-cpu 16 -min_allele_freq 0.15	-min_nbr_sites 1	-exclude_bed hg38	-mincov 4	-maxcov 160	-nbr_t '0.3,0.6'`*

Users can replace the reference file (specified by “-ref”), and the input BAM file (specify by “-bam”) for different datasets with snp or indel calling or both (specified by “-mode”). The well-trained models for SNP and indel calling can be specified by “-snp_model” and “-indel_model” parameters.

3. Please define in the paper what your size range is for an indel. 1-50bp is fairly common, and I suspect is the definition used by GIAB, but it's good to be explicit. With long-read sequencing, the line between small indels and structural variant indels is blurring.
**Authors’ Response**: Thanks for this comment. We agree with you that the difference between small indels and SVs is blurring in long-read sequencing. NanoCaller does not filter any indels according to lengths. But based on your suggestion, we add a clear statement that the threshold could be 50bp(Page 23 of main manuscript). The reason is that for the indel prediction on HG002 ONT reads, 0.023% of indels are longer than 50bp, while on CCS HG002 data, 0.59% of indels are longer than 50bp. Thus, the vast majority of predicted indels are <50bp long. Meanwhile, the benchmark v4.2.1 of HG002 variants contains 494630, 30820 and 19 indels of lengths 2-10bp, 11-50bp and >50bp respectively in high-confidence regions, with the majority of benchmark indels being <50bp long.

4. Further on this topic, could you please comment in the paper on how NanoCaller performs on indels based on their size? How large of a variant can it call? Would it work with the recently-published GIAB SV calls for hg0002?
**Authors’ Response**: Thanks for this comment. To address your comment, (1) we make a simple statistics for the indel prediction on HG002 as we discussed in the previous response: with ONT HG002 reads, 97.85% indels are 2-10bp long, 2.12% if indels are 11-50bp long, and 0.023% of indels are longer than 50bp with longest indel being 117bp long, while for CCS HG002 data 88.99% indels are 2-10bp long, 10.42% if indels are 11-50bp long, and 0.59% of indels are longer than 50bp with longest indel being 154bp long. (2) We evaluate the indel performances in high-confidence region for three indel length ranges, and find that for ONT reads, NanoCaller is able to detect 238449 out of 494565 (recall 48.21%) of 2-10bp indels, 20848 out of 30813 (recall 67.67%) of 11-50bp indels, and 1 out of 19 indels longer than 50bp. For CCS reads, NanoCaller is able to detect 449071 out of 494565 (recall 90.80%) of 2-10bp long indels, 27833 out of 30813 (recall 90.33%) of 11-50bp indels, and 4 out of 19 indels longer than 50bp. (3) We check how many indels longer than 50bp called by NanoCaller are within 100bp of an SV from HG002 SV benchmark (for SVs shorter than 200bp). We find that on ONT reads, 574 out of 901 indels longer than 50bp predicted by NanoCaller are in the benchmark SVs, whereas on CCS reads 2837 out of 5794 indels longer than 50bp predicted by NanoCaller are in the benchmark SVs. Please note that NanoCaller is not designed to call SVs, and we have been working on the development of SV caller to extend NanoCaller.

5. The ONT data used in this publication was basecalled using Guppy 2.3.x. Guppy has since undergone two major upgrades to its accuracy, in 3.6.0 and again in 4.4.0 with the introduction of the Rerio/Bonito models. While it would be too much to ask for all of the analysis in this paper to be re-run (especially since it was part of the PrecisionFDA challenge), I would like to see how NanoCaller performs on the newer, more-accurate basecalls, at least for one data set. ONT provide a public data set for hg0002, which I believe could be used for this purpose: https://nanoporetech.github.io/ont-open-datasets/gm24385_2020.09/
**Authors’ Response**: Thank you very much for this suggestion. We completely agree with you that Guppy has improved a lot in the last 2 years, and this is likely reflected in basecalling and variant calling performance. We thus download the new basecalled datasets (Please refer to Page 18 of main manuscript and Tables S28 and S29 of ‘Supplementary Materials 2’ for details), and train and test all NanoCaller models on the new basecalled data. We also performed basecalling ourselves on data sets whose Guppy 4 results are not available (Nanopore signals need to be available for us). As you have predicted, we find that NanoCaller has great improvement in both SNP and indel calling. For example, on ONT HG002 Guppy3.6 basecalled reads, NanoCaller achieved 97.99% F1-score for SNPs and 79.74% F1-score for indels in non-homopolymer regions, as shown in Table S26 of ‘Supplementary Materials 1’. When we tested NanoCaller on Bonito 0.30 basecalled reads of HG002, we obtained 99.34% F1-score for SNPs, 86.12% indel F1-score for indels in non-homopolymer regions. Details of NanoCaller and other variant callers’ performances on official data release of HG002 can be found in Tables S35 and S36 in ‘Supplementary Materials 2’ and Page 7/Table 2/Figure 3 of main manuscript.

5. The GIAB calls make for excellent training data, but raise the issue of maintaining a train/test split when evaluating software. To this end you have split your models into NanoCaller1 and NanoCaller2 for Oxford Nanopore. The authors of clair did something similar, but they also trained a model on all of the GIAB data available, for general use on non-GIAB data. Could you please explain why you have not done this?
**Authors’ Response**: Thanks for this suggestion. According to your suggestion, we have trained an ONT and a CCS model on all of the GIAB data (HG001, HG002, HG003 and HG004). We have noted that this model is for users’ general use, and it should not be used to test performance on GIAB data (HG001, HG002, HG003 and HG004).

6. Small variant calling has two major use cases in human health: resolving hereditary/germline traits, and calling driver mutations in cancer. The GIAB data makes for an excellent training and testing set for the first case. However, there is also a need for small variant callers for long-read sequence data from tumours. This data poses challenges for variant calling, including clonal heterogeneity and variable tumour content in the samples sequenced. Could you please comment in the paper on the appropriateness (or not) of NanoCaller for tumour small variant calling?

**Authors’ Response**: This is a great point and thanks for pinpointing it out. In page 16 of main manuscript, we discussed that one of advantages of NanoCaller is to easily generate multi-allelic variant calls, where all alternative alleles differ from the reference allele. Although this provides an opportunity for NanoCaller to call somatic multi-allelic variants in tumor samples with clonal heterogeneity and variable tumor content, NanoCaller is designed to call diploid alleles. Meanwhile, the frequency of some somatic variants in tumor samples might be too low to be distinguished from noises in long reads. Therefore, the analysis of tumor samples needs a careful design and parameter tuning if NanoCaller is used. Meanwhile, better performance may be achieved for a specific training model when more tumor data with truth data sets are available. We added this discussion in Page 17.

**2^nd^ round**

**Reviewer 2**

I have one additional comment: I notice in the comparisons (Table 2 and 3), that you only compared to DeepVariant on PacBio CCS data. I know that DeepVariant was included for ONT data in the PrecisionFDA Challenge, and performed well there. Could you please also show those numbers? Ideally I would like to see a comparison to the current version of PEPPER-margin-deepVariant, but just the PrecisionFDA results should suffice.

And unrelated to the manuscript itself, I would recommend including a description of the models available in the readme of the GitHub repository. I could find them after searching through the code, but it would be better to see them described there.

Thanks!

**Authors’ response**

Reviewer #2: I have one additional comment: I notice in the comparisons (Table 2 and 3), that you only compared to DeepVariant on PacBio CCS data. I know that DeepVariant was included for ONT data in the PrecisionFDA Challenge, and performed well there. Could you please also show those numbers? Ideally I would like to see a comparison to the current version of PEPPER-margin-deepVariant, but just the PrecisionFDA results should suffice.

And unrelated to the manuscript itself, I would recommend including a description of the models available in the readme of the GitHub repository. I could find them after searching through the code, but it would be better to see them described there.

**Authors’ Response**: Thanks for this comment. DeepVariant's github repository still says that DeepVariant supports Illumina and PacBio HiFi or the combination, but by itself does not support Nanopore sequencing data. It suggested users to run PEPPER (a bioRxiv print is available) first, followed by DeepVariant. Results from the DeepVariant in the PrecisionFDA Truth challenge V2 is in fact generated by this pipeline, not by DeepVariant itself.

To fully address the reviewer’s comments, we ran PEPPER-DeepVariant on ONT data basecalled with Guppy v4.2.2 for HG002, HG003 and HG004, and then evaluated the SNP/indel calling against benchmark v4.2. We now included the comparative analysis in S42 in “Supplementary Materials 2” to ensure the comparability of the results. (All results in “Supplementary Materials 2” were evaluated on Guppy v4.2.2 reads against v4.2 benchmark.) We found that NanoCaller performs better on the MHC regions, which is consistent with the original PrecisionFDA results (generated on Guppy v3.6 data over a year ago).

Additionally, we made changes to the GitHub repository to include a description of the various models that are trained to help users understand the method better. Thank you very much for the suggestions.

**3^rd^ round**

**Reviewer 2**

Thanks to the authors for the inclusion of the PEPPER-DeepVariant results. I am satisfied that my comment has been addressed.
